# Pulsed‐Laser Ablation for the Synthesis of High‐Entropy Alloy Aerogels Toward H_2_O_2_ Production and Water Decolorization

**DOI:** 10.1002/anie.2026722

**Published:** 2026-06-30

**Authors:** Cui Wang, Varatharaja Nallathambi, Lingwei Wang, Johannes Kresse, Natalia F. Shkodich, Michael Farle, René Hübner, Alexander Eychmüller, Sven Reichenberger, Stephan Barcikowski, Bin Cai

**Affiliations:** ^1^ School of Chemistry and Chemical Engineering Shandong University Ji'nan China; ^2^ Physical Chemistry Technische Universität Dresden Dresden Germany; ^3^ Technical Chemistry I and Center for Nanointegration Duisburg‐Essen (CENIDE) University of Duisburg‐Essen Essen Germany; ^4^ Max Planck Institute for Sustainable Materials Düsseldorf Germany; ^5^ Faculty of Physics and Center for Nanointegration Duisburg‐Essen (CENIDE) University of Duisburg‐Essen Duisburg Germany; ^6^ Helmholtz‐Zentrum Dresden‐Rossendorf, Institute of Ion Beam Physics and Materials Research Dresden Germany

**Keywords:** aerogels, electrosynthesis of H_2_O_2_, high entropy alloys, laser ablation, water treatment

## Abstract

Electrosynthesis of H_2_O_2_ is attractive for its environmental sustainability and cost‐effectiveness, yet is impeded by the sluggish reaction kinetics and low selectivity triggered by the competing 4e^−^ pathway. Here, a model transition‐metal‐based multimetallic aerogel was designed using CrMnFeCoNi HEA nanoparticles from nanosecond‐pulsed laser synthesis in liquids, along with three exemplary quaternary systems without Co, Fe, and Ni, respectively. Among them, the resulting CrMnFeCoNi HEA aerogel exhibits the highest H_2_O_2_ selectivity of 95% and the lowest transferred electron number of 2.1, as well as good stability of nearly 100% H_2_O_2_ selectivity after 10k cycles. Furthermore, the as‐prepared CrMnFeCoNi aerogel reaches a maximum H_2_O_2_ yield of 2.34 mmol h^−1^ $cm_{disk}^{-2}$ and demonstrates an efficient decolorization ability for organic pollutants (e.g., Methylene blue or Rhodamine B). This outstanding performance is attributed to the synergetic effects of the various metals and the configurational entropy contribution, enabling a favored distribution of surface atom arrangements and optimal binding energies during electrochemical reactions. This work not only provides a novel perspective for manipulating HEA aerogels but also presents a promising alternative for industrial H_2_O_2_ production and water treatment.

## Graphical abstract

This work establishes a universal strategy for fabricating compositionally complex solid solution alloy aerogels via pulsed‐laser ablation and the sol‐gel process. The electrocatalytic capabilities of these aerogels are subsequently evaluated for H_2_O_2_ electrosynthesis and water treatment applications. Among them, the as‐synthesized CrMnFeCoNi high‐entropy alloy aerogel shows the most outstanding performance, attributed to an optimally distributed surface atomic configuration, a slight enrichment of Mn on the surface, and collective interactions of Fe, Co, and Ni.

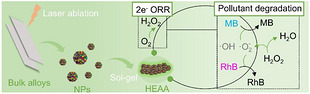

## 1 Introduction

H_2_O_2_ is a crucial industrial commodity with degrading properties on dyes and antibiotics, as well as disinfecting and sterilizing effects [1, 2, 3, 4]. Currently, the anthraquinone process is a universal route for H_2_O_2_ commercial production, suffering from intense energy consumption and potential environmental and safety risks. The direct electrosynthesis of H_2_O_2_, without additives or reaction cycles as required in the anthraquinone process, has attracted considerable attention for its superior environmental sustainability and cost‐effectiveness [3, 5, 6, 7, 8]. Moreover, leveraging renewable electricity enables the flexible use of low‐cost power during periods of high availability and low demand, reducing costs for on‐site H_2_O_2_ production for processes with moderate H_2_O_2_ consumption. Hence, this approach seamlessly integrates with electro‐Fenton or Fenton‐like reactions, facilitating the efficient degradation of environmental pollutants. 2e^−^ oxygen reduction reaction (ORR) is widely recognized as a promising alternative for electrochemical H_2_O_2_ production [1, 9]. However, the sluggish reaction kinetics and low selectivity triggered by the 4e^−^ competitive approach impede its development and commercial viability [10, 11]. Therefore, it is desirable to rationally manipulate and optimize electrocatalysts to realize outstanding selectivity for the electrosynthesis of H_2_O_2_.

Recently, high‐entropy alloys (HEA) or compositionally complex solid solution (CCSS) alloys have become in vogue because they exhibit exceptional properties exceeding those of conventional unary or binary systems in various fields, including catalysis [12, 13, 14, 15]. Multiple principal elements (five or more) combined in near equal proportions form a solid solution promoted by entropic stabilization, giving rise to the unique combination of characteristics like sluggish diffusion, lattice distortion, and the resulting cocktail effects [16, 17, 18, 19, 20]. Particularly for electrocatalysis, the presence of several different near‐neighbor atoms on the surface enables tailoring the binding energies of reaction intermediates (and hence reaction selectivity) depending on the composition of the constituent elements [21, 22, 23, 24]. Many experimental and computational studies reported better electrocatalytic activities and performance of HEAs compared to single‐element catalysts [25, 26, 27, 28, 29, 30, 31, 32, 33] and the possibility of substituting expensive and rare platinum‐group elements with more earth‐abundant elements [28, 34, 35, 36, 37, 38]. Yet, the synthesis of nanoparticulate HEA catalysts with uniform element distributions remains challenging. Different synthesis methods [39, 40], ranging from solvothermal, colloidal, electrodeposition, template‐assisted, photo‐thermal, mechanical alloying, and others, have been attempted to prepare HEA nanomaterials and study their structure‐activity relationships [18, 41, 42, 43]. However, the local surface structure and composition distribution of the resulting HEA depend heavily on the synthesis method and chosen HEA composition, as well as whether the HEA nanoparticles (NPs) were self‐standing or substrate‐supported. Among these established synthesis methods, pulsed‐laser ablation in liquids (PLAL) represents a rather unique approach that is scalable, reaching multiple g h^−1^ productivity [44, 45], while prepared HEA nanoparticle composition series showed distinct composition‐activity correlations [46, 47, 48, 49]. PLAL therefore represents a viable synthesis pathway, offering key advantages such as compositional tunability for broader exploration of the HEA composition space, as well as surfactant‐free colloidal nanoparticles that can be easily deposited onto conductive substrates or used as self‐supporting aerogel structures. Hereby, PLAL typically yields electrostatically stabilized colloidal nanoparticles in a chosen liquid, where the nanoparticle composition is nearly identical to the composition of the target that is being used in the ablation [44, 45]. The latter is particularly linked to the involved rapid cooling conditions (>1000 K ns^−1^ during the first ns after laser‐induced heating of the target) that occur on pico‐ and nanosecond time scales, while the target is locally being heated by a short‐ or ultrashort laser pulse but also in caloric contact with the surrounding liquid [50, 51, 52]. Hence, although PLAL is practically executed at room temperature and ambient pressure, locally, at the microscopic laser pulse impact site, it is not. These rapid temperature gradients are thought to greatly aid the formation of complex solid solutions in the generated colloidal HEA NPs [53, 54], an advantage over the chemical synthesis methods that are prone to poor mixing or phase segregation (unless executed at high temperature and with bulky precursor molecules). Aerogels, self‐assembling from nanoparticles via the typical “two‐step” approach, can prevent the uncontrolled aggregation of nanoparticles and (the inevitable) corrosion of the carbon support. To exploit the synergies of both methods, a combined approach between kinetically controlled PLAL and nanoparticle gelation is implemented to produce HEA aerogels. HEA aerogels, leveraging the advantage of aerogels with high porosity and self‐supported structure, are considered promising alternatives in electrocatalysis. Realizing equimolar multimetallic alloy aerogels with a stable single‐phase structure presents significant challenges, compounded by the high cost of precious metals and the limited research into their potential for H_2_O_2_ electrocatalysis. These facts underscore the urgent need for intensified research efforts in this area.

In this work, we address the above‐mentioned challenges regarding the pulsed laser ablation strategy for the regulation of HEA aerogels with a model system based on the constituents of the well‐known Cantor alloy CrMnFeCoNi, along with three exemplary quaternary systems without Co, Fe, or Ni, respectively. The HEA nanoparticles were first fabricated from different bulk targets by PLAL (water, ethanol, and acetone) with similar chemical composition, followed by the evaluation of the effect of solvent permittivity on the porosity of the resulting aerogels. Under the optimal gelation condition, the resulting CrMnCoNi, CrMnFeNi, CrMnFeCo, and CrMnFeCoNi aerogels were used for the electrosynthesis of H_2_O_2_ and water treatment. Among them, the as‐prepared CrMnFeCoNi aerogel from NPs synthesized in water exhibits the highest H_2_O_2_ selectivity of 95% and the lowest transferred electron number of 2.1, as well as good stability of nearly 100% after 10k cycles, outperforming the other three CrMnCoNi, CrMnFeNi, and CrMnFeCo aerogels. The reason behind this impressive performance can be attributed to a slight enrichment of Mn on the surface, synergetic effects from Fe, Co, and Ni, and optimal binding energies through electrochemical reactions. Additionally, the as‐prepared CrMnFeCoNi aerogel was further employed for practical H_2_O_2_ production via an H‐cell and water treatment for organic pollutants (e.g., Methylene blue (MB) or Rhodamine B (RhB)), where it reaches a maximum H_2_O_2_ yield of 2.34 mmol h^−1^ $cm_{disk}^{-2}$ at an optimal working potential of 0.2 V_RHE_ and demonstrates the efficient decolorization ability for organic pollutants. This work not only provides a new perspective for manipulating HEA aerogels but also a promising alternative for industrial H_2_O_2_ production and water treatment.

## 2 Results and Discussion

### 2.1 Synthesis and Characterization of High‐Entropy Alloy Aerogels

A schematic of the “two‐step” approach employed to synthesize HEA solvogels, in which the HEA NPs are directly generated from the corresponding bulk targets via pulsed laser ablation in a liquid, followed by the gelation process triggered by the salting‐out effect, is shown in Figure S1a [55]. The detailed phase structure and composition characterization of the source materials prepared using high‐energy ball milling for target preparation can be found in the Supporting Information. The solvents used during the laser ablation synthesis have an essential impact on the surface chemical environment, thus tailoring the properties of the resulting NPs [56, 57] and hence the aerogels. Herein, CrMnFeCoNi NPs were synthesized via PLAL in water, ethanol, and acetone. Subsequently, the colloidal nanoparticles were gelated via a typical two‐step gelation approach and characterized via TEM. Three different solvents were chosen for the initial PLAL synthesis step to leverage the effect of particle‐solvent interaction during laser synthesis [57]. An aqueous environment is known to typically result in a mild surface oxidation of the produced NPs, while the ablation in ethanol and acetone reduces the degree of oxidation [57]. Additionally, laser‐induced decomposition of organic solvents can result in carbon shell formation around the NPs. More background information can be found in Ref. [54].

**FIGURE 1:**
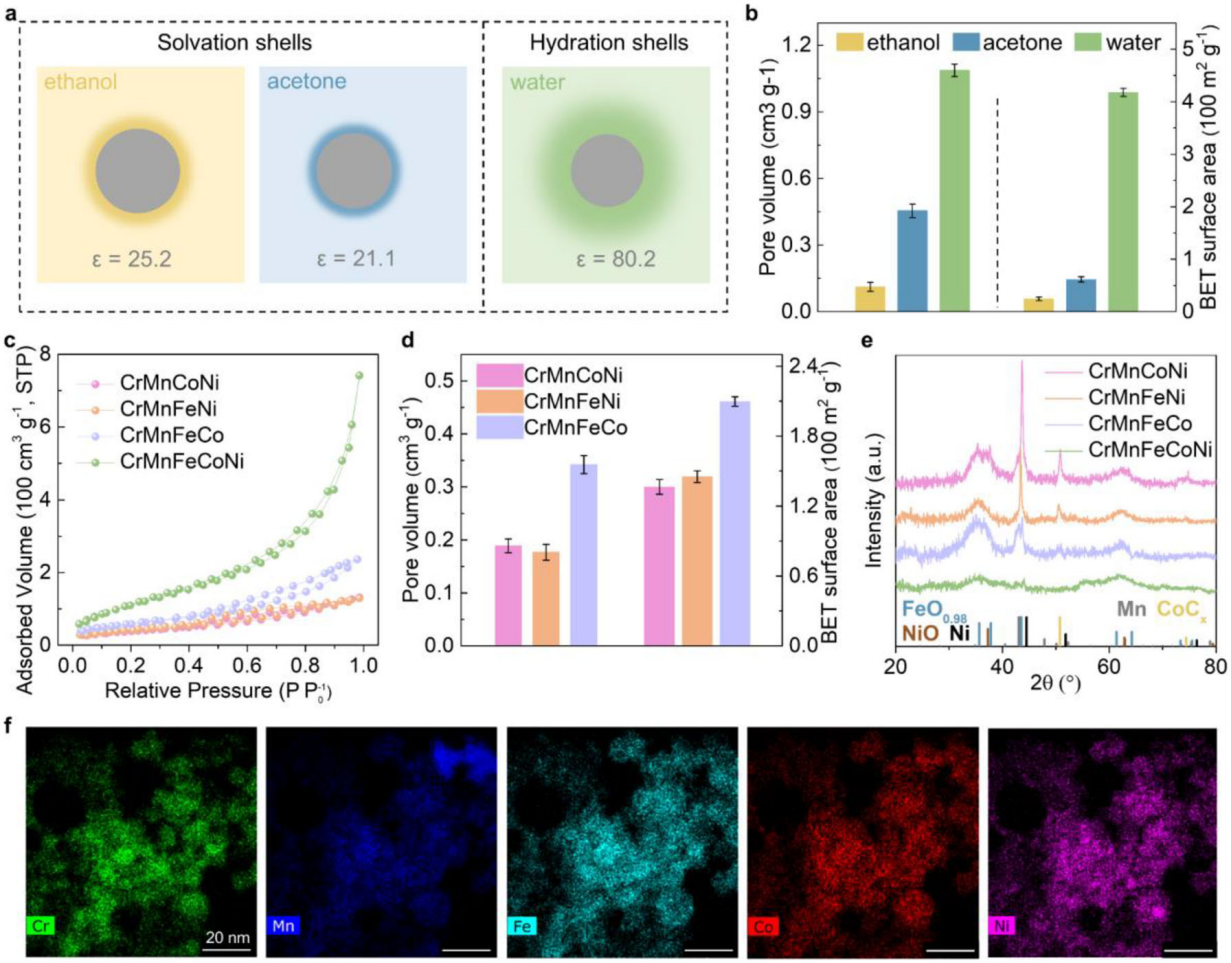
Synthesis and characterization of MEA and HEA aerogels. (a) Scheme of the resulting HEA NPs with solvation or hydration shells, obtained from laser‐ablation toward the bulk materials in ethanol (yellow shell), acetone (blue shell), and water (green shell). The diameter of the depicted greyish circles (which represent the HEA NPs) reflects the particle diameters from Figure S1, where NPs prepared in water were taken as a benchmark. ε represents the corresponding dielectric constant of the solvent [3]. (b) Pore volume (left) and BET surface area (right) of the resulting CrMnFeCoNi HEA aerogels obtained in different solvents. (c) N_2_ desorption and absorption curves of MEA and HEA aerogels with different compositions. (d) Pore volume (left) and BET surface area (right) of the resulting three MEA aerogels obtained in water. (e) XRD patterns of the four indicated aerogels, subscrated background from Figure S5. (f) EDS‐based element distribution maps of Cr, Mn, Fe, Co, and Ni for the as‐prepared CrMnFeCoNi aerogel. Error bar in Figure 1b,d represent standard deviation from three‐time measurements.

Representative TEM images and respective particle size distributions from analysis of the geometric particle diameter of around 100 particles gained in the respective solvents are summarized in Figure S1b–f. As can be seen, the average number‐weighted geometric diameters of the primary particles in the aerogels are 35 ± 20, 31 ± 17, and 30 ± 14 nm for ethanol, acetone, and water, respectively (Figure S1b–f), showing comparable size distributions. As schematically depicted in Figure 1a, the solvation shells of the NPs obtained in ethanol (dielectric constant, ε  =  25.2) are slightly larger than those in acetone (ε  =  21.1), implying a positive correlation between the dielectric constant and the size of the solvation shell [58, 59]. For water, the largest hydration shell and stronger colloid stability can be observed due to the highest dielectric constant (ε  =  80.2) [58]. These variations of the surface state of building blocks can lead to differences during the formation of the resulting aerogels [60]. Compared to the solvogels obtained in ethanol and acetone (∼5 h), the CrMnFeCoNi HEA hydrogel fabricated in water requires a longer gelation period (∼12 h) due to the more pronounced electrostatic stabilization [59, 61]. In addition to TEM, the pore‐size distributions and Brunauer–Emmett–Teller (BET) surface areas of the respective aerogels were characterized via nitrogen physisorption isotherms. (Figures 1b and S2). In particular, the BET surface area of the CrMnFeCoNi HEA aerogel fabricated in water reaches up to 417.8 m^2^ g^−1^, which is 17.1 and 6.7 times higher than those in ethanol and acetone, respectively, emphasizing the significance of the surrounding medium and building block surface state. Moreover, this BET surface area is remarkably increased by an order of magnitude compared to that of pure noble metal aerogels [4], which can be attributed to the lower relative molecular mass (and density) of ignoble metals and the presence of surface oxides (see below) [62].

In order to investigate the importance of the multi‐element environment in the HEA NPs, three medium‐entropy alloy (MEA) aerogels (CrMnCoNi, CrMnFeNi, and CrMnFeCo), which represent the HEA composition but without Fe, Co, and Ni, respectively, were prepared. The synthesis procedure of the MEA aerogels was identical to the HEA ones except for the different bulk target compositions, with the laser ablation only done in water. As can be seen from Figures 1c,d and S3 and S4; Table S1, the MEA aerogels were obtained with comparable particle sizes, pore radius distributions, and BET surface areas.

X‐ray diffraction (XRD) analysis was done to determine the phase composition of the as‐prepared HEA and MEA aerogels (see Figure 1e and Figure S5). Apparently, a broad diffuse diffraction maximum in the 2θ range of 5–30° are explicitly observed (Figure S5), indicating a possible contribution from an amorphous phase in the as‐prepared aerogels. In addition, the peaks at around 42° and 50° represent the formation of an fcc phase in the resulting aerogels, which is in agreement with a previous work on HEA NPs [48]. The presence of an amorphous phase as well as the synthetic effect of Fe, Co, and Ni, is expected to enhance the electrocatalytic properties due to an enhanced density at coordinately unsaturated sites and a metastable state with higher Gibbs free energy in the amorphous structure [63, 64]. In addition, there are some low‐ or high‐intensity peaks located at around 38° and 45°, implying the possible presence of secondary phases [48]. Spectrum imaging analysis based on energy‐dispersive x‐ray spectroscopy (EDS) performed in scanning transmission electron microscopy (STEM) mode was conducted to validate the element distributions in the as‐prepared aerogels. Figures 1f and S6–S8 suggest that all elements (Cr, Mn, Fe, Co, and Ni) from the bulk target are present in the resulting aerogels and show rather homogeneous distributions. Dissolved oxygen molecules in the reaction medium (i.e., water) led to the emergence of surface oxides in line with previous studies [48, 65].

**FIGURE 2:**
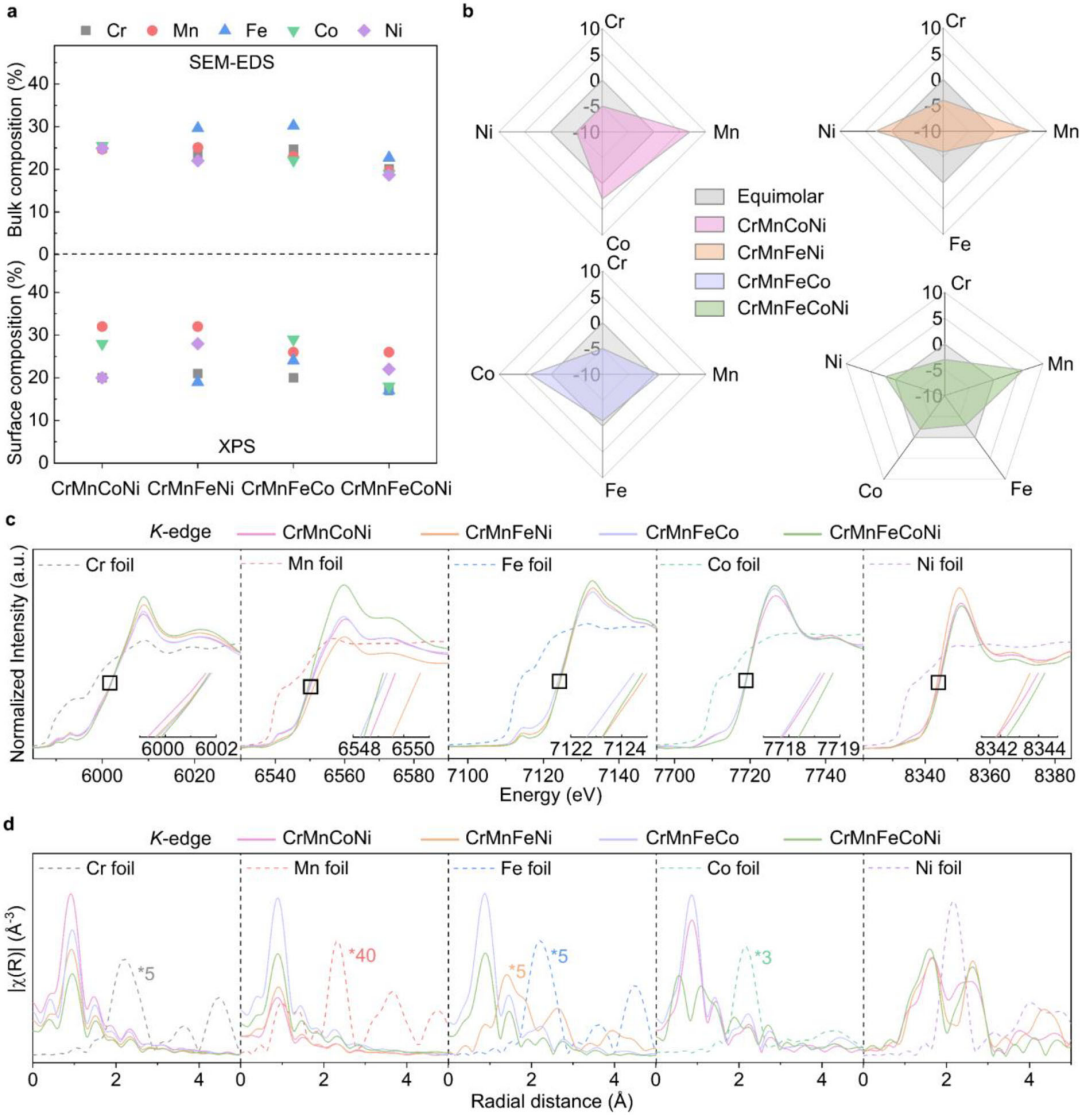
Compositional and structural analysis of the HEA and MEA aerogels. (a and b) Compositional analysis. (a) Relative bulk element compositions from SEM‐EDS measurements (top) and XPS surface element compositions calculated from the high‐resolution XPS spectra of the Cr 2p, Mn 2p, Fe 2p, Co 2p, and Ni 2p peaks (bottom). (b) Deviation of the surface element composition from the ideal equimolar concentration corresponding to the four aerogels. (c and d) X‐ray absorption spectroscopy analysis. (c) Normalized K‐edge XANES spectra of Cr, Mn, Fe, Co, and Ni for the metal foils and the as‐prepared aerogels. (d) k^2^‐weighted Fourier transforms (R space) of the EXAFS spectra derived from (c). The insets in (c) show magnified views of the regions indicated by squares.

The relative bulk element compositions of the as‐prepared aerogels measured using SEM‐EDS analysis are shown in Figure 2a (top) and Table S2. The individual element concentrations of the CrMnFeNi and CrMnFeCo compositions are close to the expected equimolar ratios, with only a slight enrichment of Fe in the aerogels similar to their starting micropowders (see Figures S9–S13 and Table S3). Furthermore, the multimetallic aerogels were characterized by x‐ray photoelectron spectroscopy (XPS) to determine the surface element distributions. The 2p spectra of the constituent elements were recorded, and the deconvoluted XPS peaks indicated the presence of metallic species in addition to surface oxide/hydroxide species. The relative surface element compositions calculated from the high‐resolution XPS spectra of the Cr 2p, Mn 2p, Fe 2p, Co 2p, and Ni 2p peaks of all four HEA and MEA aerogels are shown in Figure 2a (bottom), which is consistent with inductively coupled plasma (ICP) analysis (Table S4). Detailed information on the peak fitting parameters and representative fitted spectra can be found in Table S5 and Figures S14–S17, respectively. A slight enrichment of Mn on the surface compared to other elements can be found, which results from the preferential oxidation during laser‐ablation synthesis in water medium. Since near‐equimolar concentrations result in the highest configurational entropic contribution, the deviation of the surface element composition from the ideal ratio of each system is highlighted in Figure 2b.

X‐ray absorption spectroscopy was employed to unravel the local electronic and structural environment [66] of the as‐prepared HEA and MEA aerogels. The K‐edge x‐ray absorption near‐edge structure (XANES) spectra of the aerogels deviate systematically from the corresponding metal foils (Figure 2c). In particular, Mn exhibits the most pronounced positive shift of the white‐line (E_WL_) position relative to the Mn foil (∆E_WL_ = +2.5 to +3.0 eV) among all elements, together with an overall enhancement of the white‐line intensity (Table S6 and Figure S18). This phenomenon is consistent with preferential oxidation (or likely hydroxylation under aqueous conditions, or a more electron‐deficient environment) at Mn sites [67, 68, 69]. In contrast, Co shows an opposite shift of the white‐line position (∆*E*_WL_ = −2.46 to −2.87 eV) despite an increased white‐line intensity, suggesting that Co undergoes a different type of local electronic and coordination reorganization rather than a simple rigid edge shift (Table S6 and Figure S18). The Fourier Transform (FT)‐EXAFS magnitudes (χ|(R)|) display strongly attenuated higher‐shell contributions relative to the foils (Figure 2d), consistent with predominantly short‐range order in the aerogel frameworks [70]. Additional low‐R features observed for most elements suggest the presence of M─O coordination [66], while the Ni K‐edge FT shows a distinct doublet, indicative of coexisting local environments (e.g., Ni─O and Ni‐metal coordination) in the as‐prepared aerogels [71]. These structural motifs are corroborated by XPS, which reveals an increased fraction of oxidized (hydroxylated) surface components for CrMnFeCoNi compared with the quaternary aerogels. Taken together, the as‐prepared aerogels are consistent with a mixed local coordination environment, which may contribute to different electrochemical behavior toward H_2_O_2_ generation.

### 2.2 Electrocatalytic H_2_O_2_ Production

**FIGURE 3:**
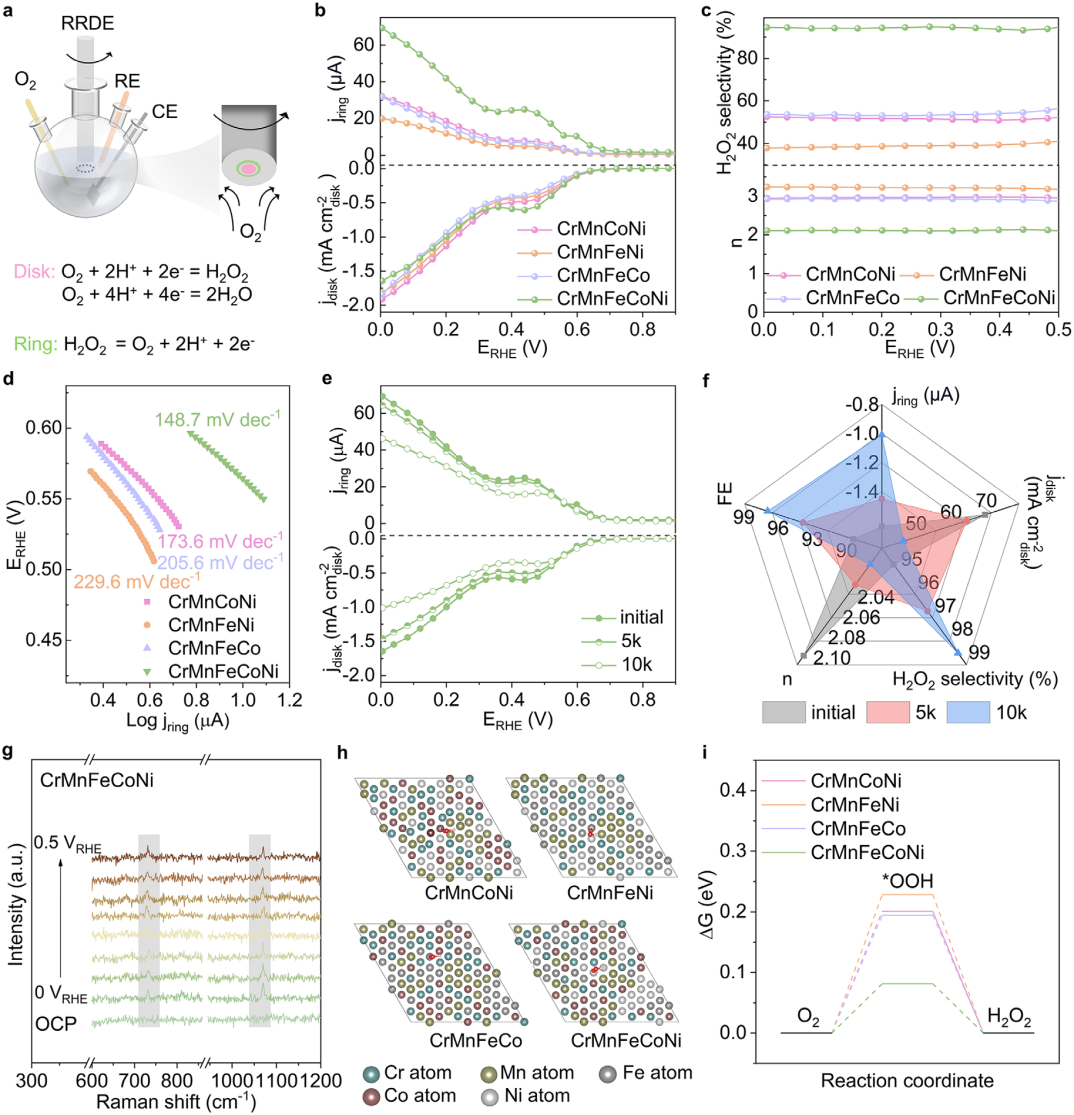
Electrocatalytic H_2_O_2_ production performance. (a) Scheme of electrocatalytic 2e^−^ oxygen reduction reaction (ORR) for H_2_O_2_ production conducted on a rotating ring‐disk electrode (RRDE). (b) RRDE polarization curves (bottom) and detected H_2_O_2_ currents at the ring electrode (top), (c) calculated H_2_O_2_ selectivity (top) and transferred electron numbers (denoted as n, bottom), and (d) Tafel slopes of the resulting multimetallic aerogels. (e) RRDE polarization curves (bottom) and detectable H_2_O_2_ currents at the ring electrode (top) and (f) corresponding performance parameters of the resulting CrMnFeCoNi aerogel after different cycles of the accelerated durability test (ADT). (g) In situ Raman spectra recorded over the CrMnFeCoNi aerogel at various applied potentials (0 V_RHE_, 0.1 V_RHE_, 0.2 V_RHE_, 0.25 V_RHE_, 0.3 V_RHE_, 0.35 V_RHE_, 0.4 V_RHE_, and 0.5 V_RHE_), whereas OCP represents open circuit potential. (h) The thermodynamically favorable atomic configuration of the multimetallic aerogels with ^*^OOH absorbed on the (111) facet. (i) Free energy diagram of 2e^−^ ORR at different catalysts.

Motivated by their distinctive structure and property, the resulting multimetallic aerogels were developed for the 2e^−^ oxygen reduction reaction (ORR) in O_2_‐saturated 0.1‐M KOH medium, where the ORR took place at the disk electrode and the generated H_2_O_2_ was subsequently oxidized at the ring electrode (Figures 3a and S19 and S20) [5]. Figure 3b shows the linear sweep voltammetry (LSV) curves of different aerogels on the disk electrode, indicating a subtle difference in their current densities at 0 V_RHE_ (−1.75 ± 0.15 mA $cm_{disk}^{-2}$). However, a considerable difference can be observed in their H_2_O_2_ oxidation current (Figure 3b and Table S7). The ring current density of the CrMnFeCoNi aerogel reaches up to 70 µA at 0 V_RHE_, which is 2‐ and 3.5‐fold higher than that of the CrMnFeCo (or CrMnCoNi) and CrMnFeNi aerogels, respectively. Additionally, in a wider range of 0–0.6 V_RHE_, the ring current of the CrMnFeCoNi aerogel significantly surpasses that of the other catalysts, meaning an outstanding H_2_O_2_ generation ability. Based on the disk and ring currents, the H_2_O_2_ selectivity and the transferred electron numbers were calculated (Figure 3d,e). The resulting CrMnFeNi aerogel possesses a minimum selectivity of 38.0% and a maximum transferred electron number of 3.2, illustrating the competition in 2e^−^ and 4e^−^ ORR [72]. The other two aerogels (i.e., CrMnCoNi and CrMnFeCo) exhibit an analogous electrocatalytic behavior, endowed with a moderate H_2_O_2_ selectivity of 53.9% and a transferred electron number of 3.0. Notably, at the co‐existence of Fe, Co, and Ni, the as‐prepared CrMnFeCoNi aerogel shows the highest H_2_O_2_ selectivity of 95.0% (Figure 3c), a Faradaic efficiency (FE) of 90% in Figure 3f, and the lowest transferred electron number (n) of 2.1 (experimental details how the FE and n were determined see experimental section in the Supporting Information), implying a remarkable 2e^−^ ORR activity compared to most of the current catalysts (Table 1). Moreover, the reaction kinetics were determined by the corresponding Tafel slopes (Figure 3c). Among the catalysts, the CrMnFeCoNi HEA aerogel exhibits the smallest Tafel slope of 148.7 mV dec^−1^, demonstrating the fastest reaction kinetics [5].

**TABLE 1:** Comparison of the 2e^−^ ORR performance of different catalysts.

Catalysts	Electrolytes	H_2_O_2_ selectivity (%)	Transferred electron numbers	Ref.
ZnCo‐ZIF	0.1 M PBS	∼92%	—	[5]
PD/N‐C	0.1 M HClO4	80%–98%	∼2	[9]
Co_2_‐DAC	0.1 M HClO4	>95%	2.1	[3]
Au‐Pd_2_Hg_5_ aerogel	0.1 M HClO4	92.8%	∼2.1	[6]
C‐MOF Ni‐250	0.1 M KOH	92%	∼2.1	[78]
NiZn MOF	0.1 M KOH	90%	∼2.1	[79]
CrMnFeCoNi aerogel	0.1 M KOH	95%	∼2.1	This work

**FIGURE 4:**
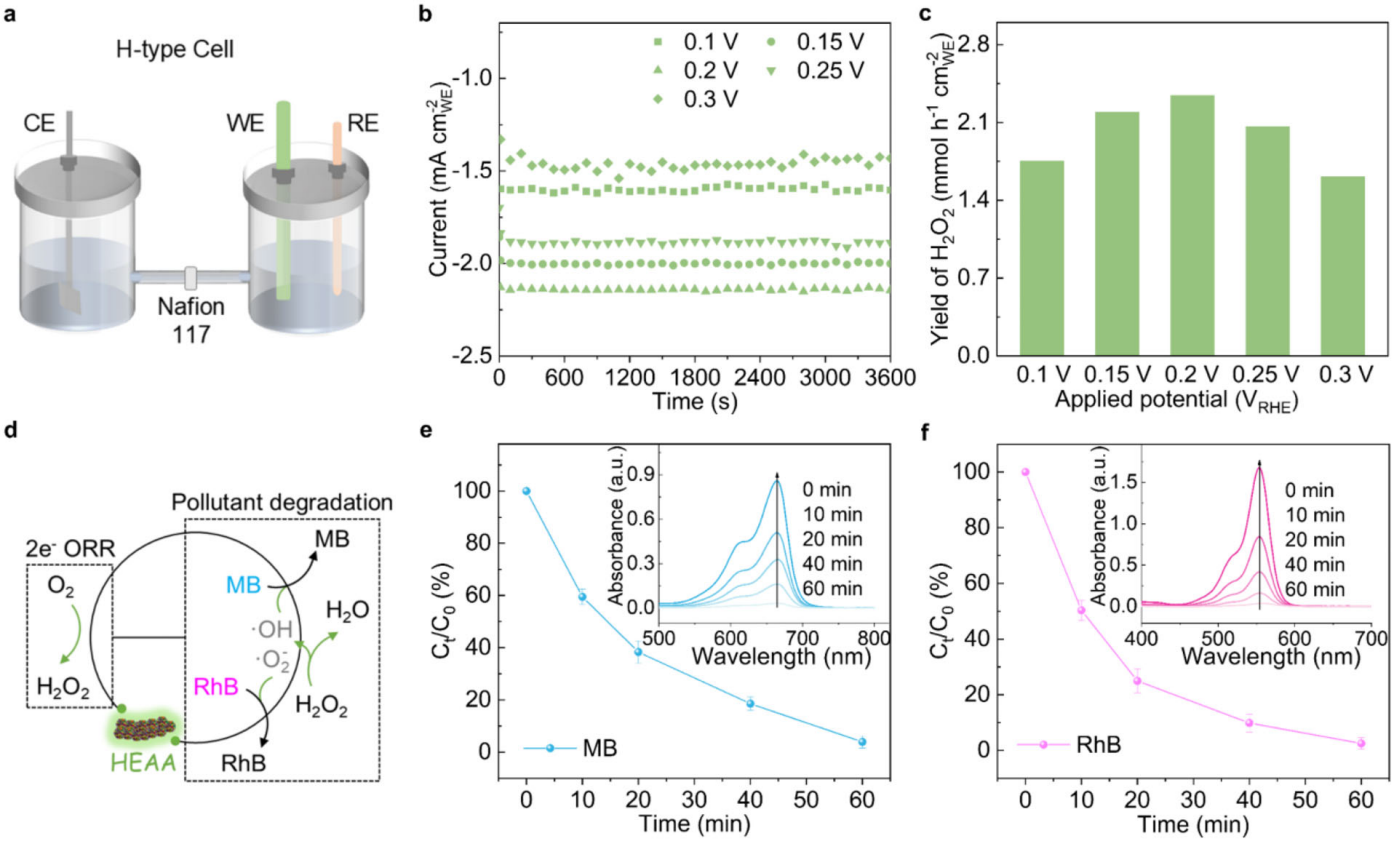
H_2_O_2_ production and water treatment. (a) Scheme of the H‐type cell used in this work. (b) Current density and (c) the corresponding yield of H_2_O_2_ of the resulting CrMnFeCoNi HEA aerogel (HEAA) at different applied potentials (V_RHE_). “WE” in the y‐axis title in (c) denotes the working electrode, which was a carbon cloth (1 cm × 1.5 cm). (d) Proposed integration reaction mechanism initiated by HEAA, including electrosynthesis of H_2_O_2_ via 2e^−^ ORR and water treatment via pollutant decolorization. (e‐f) Plots and the corresponding UV spectra (inset) of (e) MB and (f) RhB decolorization at different time.

Furthermore, the stability of the CrMnFeCoNi aerogel was evaluated by an accelerated durability test (ADT), which was performed in the potential window between 0.15 and 0.75 V_RHE_ in O_2_‐saturated 0.1‐M KOH solution at 100 mV s^−1^ (Figure 3e,f). The corresponding disk current and ring current show almost no change after 5k cycles, demonstrating eminent stability. Increasing the durability test to 10k cycles, both the corresponding disk current and the ring current show a similar tendency of attenuation, which may be induced by electrolyte deterioration [73]. Strikingly, the corresponding performance parameters (e.g., transferred electron numbers, selectivity, and FE) exhibit a shrinkage after 5k cycles but an enhanced behavior after 10k cycles. This illustrates the subtle change in the intrinsic activity of the HEA aerogel and a new equilibrium between electrolyte and catalyst [74], which is consistent with the unchanged morphology and element distribution (Figure S21). To gain insight into potential‐dependent interfacial species during ORR, in situ Raman was recorded on the CrMnFeCoNi aerogel (Figure 3g). Two additional bands at 729 and 1070 cm^−1^ were detected throughout the investigated potential range, showing only weak potential dependence with a discernible attenuation at 0.4 and 0.5 V_RHE_. This behavior is compatible with an interfacial contribution, which may be tentatively attributed to O–O‐containing surface species (e.g. ν(^*^OOH) and ν(O_2_^*^) as reported in previous work [75, 76] although isotope‐labeling validation is still needed. DFT calculations were performed to rationalize the distinct electrochemical behaviors of the as‐prepared aerogels. Since ^*^OOH is a key descriptor for the 2e^−^ ORR pathway [77], Figure 4h shows the optimized ^*^OOH adsorption configurations on the constructed multimetallic slab models. The Gibbs free energy diagram for the 2e^−^ ORR pathway (Figure 4i) indicates that the CrMnFeCoNi slab model possesses ^*^OOH adsorption energetics closest to thermoneutrality, with a calculated |∆G_*OOH_| value of 0.08 eV. This value is substantially smaller than those calculated for the other multimetallic slab models, suggesting more balanced ^*^OOH formation and desorption energetics on the CrMnFeCoNi model surface. The calculated trend is consistent with the experimentally observed H_2_O_2_ selectivity (Figure 4c), implying that compositional and structural regulation may contribute to tuning ^*^OOH energetics. Nevertheless, because the slab models represent idealized local surface configurations, the DFT results should be interpreted mainly as a qualitative comparison of relative energetic trends rather than a complete description of all possible active sites on the real aerogel surface.

### 2.3 Deciphering the Structure‐Composition‐Property Relationship

To further understand the surficial property of these resulting aerogels, the electrochemically active surface area (ECSA) was calculated by determining the double‐layer capacitance (C_dl_) of the system from the corresponding CV curves (Figure S22) recorded as a function of the potential sweeping speed [80]. It is found that the CrMnFeCoNi aerogel possesses the highest C_dl_ of 1.83 mF, while those for the CrMnCoNi, CrMnFeNi, and CrMnFeCo aerogels are 0.54, 0.67, and 0.97 mF, respectively (Figure S22). The substantial difference in ECSA can be ascribed to the similar morphology and the distinguished pore‐size distribution, suggesting a facilitated intrinsic activity of the CrMnFeCoNi HEA aerogel.

The interesting electrocatalytic behavior of the multimetallic alloys in terms of performance comes from the complex element environment, creating multifunctionally active sites. The better stability arises from the entropic phase stabilization, which is further supported by the lattice distortion and sluggish diffusion effects [12, 13, 21, 24]. The polyelement surface atom arrangements (SAAs) found in these materials tend to provide a continuous distribution of binding energies that can be tuned for the respective electrochemical reactions [12]. The higher degree of polyelement SAAs present on the surface enables the availability of sufficient active sites, thereby providing better electrochemical activity compared to the individual element systems. Such a statistically favored distribution of SAAs can be seen in the case of the CrMnFeCoNi aerogel from Figure 2a,e, where the concentrations of Fe, Co, Ni, and Cr are close to the nominal compositions with minimal deviations, suggesting the preferential segregation or short‐range ordering on the surface is close to none. Previous studies have shown that a slight enrichment of Mn on the surface could help to enhance the catalytic activity [46, 48]. Also, the combination of Fe, Co, and Ni being present on the SAAs of the CrMnFeCoNi aerogel is beneficial for the enhanced activity compared to the quaternary counterparts. From Figure 2a,d, the CrMnFeCo aerogel can be seen as the next‐best candidate in terms of the deviation from the nominal equimolar concentration, which reflects the second‐best activity observed next to the CrMnFeCoNi aerogel.

### 2.4 H_2_O_2_ Production and Water Treatment

Encouraged by the adequate electrochemical performance of the resulting CrMnFeCoNi HEA aerogel, a promising integrated system for water treatment (i.e., pollutant decolorization) was designed ingeniously, employing H_2_O_2_ molecules as a relay intermediate. Therefore, the performance of H_2_O_2_ production was first evaluated with the aid of an H‐type cell (Figure 4a). Herein, a Nafion 117 film was employed to connect the cathode and anode (both electrolytes are O_2_‐saturated 0.1‐M KOH) [13], and H_2_O_2_ was generated at the cathodic cell by chronoamperometric measurements. As shown in Figure 4b,c, the applied potential on the working electrode influences the current density and the corresponding yield of H_2_O_2_. Apparently, for the CrMnFeCoNi HEA and the potential range between 0.1–0.3 V_RHE,_ the recorded current densities are maintained over a duration of 1 h, which indicates a good catalytic stability (Figure 4b). When plotting the current density vs. the applied voltage (vs. RHE), a volcano‐like behavior can be observed (Figure 4c), manifesting that the maximum yield of H_2_O_2_ can reach 2.34 mmol h^−1^ $cm_{disk}^{-2}$ under the optimal working potential of 0.2 V_RHE_ (Figures 4c and S23 and S24). The good stability and the decent H_2_O_2_ production ability manifest the huge potential for practical water treatment, thereby the integrated system is constructed (Figure 4d).

In particular, in the first step, H_2_O_2_ electro‐synthesized by 2e^−^ ORR is accumulated in the cathodic solution. Subsequently, organic dyes, such as MB and RhB, can undergo decolorization in the second step in the presence of the H_2_O_2_‐containing solution [81] and the CrMnFeCoNi aerogel. As a proof‐of‐concept, the electrolyte, including H_2_O_2,_ was collected from the cathodic solution. Notably, the as‐prepared CrMnFeCoNi HEA aerogel plays an indispensable role in this decolorization step. The initial solution of MB and RhB (both of 16 mg L^−1^) displays distinctive UV adsorption at 664 nm and 554 nm [82, 83], respectively (Figure S25). It is only through the interaction of the cathode solution and the catalyst that the phenomenon of the decolorization of the pollutants can be observed, both in the dark and in ambient light (Figure S25). Within the first 10 min, the characteristic absorption intensities of MB and RhB decreased by 40.5% and 49.8%, respectively, and decolorization efficiencies of 95.9% and 97.2% were achieved after 60 min (Figures 4e,f and S26). These results suggest that the HEA aerogel can promote the activation of the H_2_O_2_‐containing cathodic solution and facilitate dye decolorization, possibly involving reactive oxygen species as reported in related systems [84].

## 3 Conclusion

In summary, a series of multimetallic aerogels was successfully fabricated by gelation of nanoparticles prepared from different bulk targets by nanosecond‐pulsed laser ablation in liquids. Among them, the as‐prepared CrMnFeCoNi aerogel exhibits the highest H_2_O_2_ selectivity of 95%, a Faradaic efficiency for H_2_O_2_ of 90%, the lowest transferred electron number of 2.1, as well as good stability of nearly 100% after 10k cycles, outperforming the other three CrMnCoNi, CrMnFeNi, and CrMnFeCo aerogels. From this comparison, we suggest that the impressive performance of the quinary system compared to the quaternary counterparts is linked to the electronic structure and surface element distributions in the CrMnFeCoNi aerogel. Hereby, it is noteworthy that Cr appeared to be slightly enriched on the surface (20.1 at% instead of 19.9% in bulk composition) for the CrMnFeCoNi composition. Additionally, the as‐prepared CrMnFeCoNi aerogel was further employed for practical H_2_O_2_ production using an H‐type cell and water treatment for organic pollutants (e.g., Methylene blue or Rhodamine B). The maximum yield of H_2_O_2_ can reach up to 2.34 mmol h^−1^ $cm_{disk}^{-2}$ at an optimal working potential of 0.2 V_RHE_, and demonstrates effective decolorization performance. Taken together, beyond expanding the synthesis and application space of HEA aerogels, this work delineates a practical pathway that could serve as an alternative for industrial H_2_O_2_ production and water treatment.

## Author Contributions

**Cui Wang**: conceptualization, methodology, validation, visualization, writing – review and editing, writing – original draft, formal analysis, data curation, investigation. **Varatharaja Nallathambi**: methodology, validation, visualization, formal analysis, data curation, writing – original draft, writing – review and editing. **Lingwei Wang**: visualization, formal analysis, data curation, writing – review and editing. **Johannes Kresse**: visualization, data curation, writing – review and editing. **Natalia F. Shkodich**: formal analysis, writing – review and editing. **Michael Farle**: writing – review and editing, supervision, resources, conceptualization. **René Hübner**: visualization, writing – review and editing, data curation. **Alexander Eychmüller**: validation, supervision, resources, methodology, funding acquisition, conceptualization, data curation, writing – review and editing. **Sven Reichenberger**: supervision, resources, methodology, data curation, conceptualization, writing – review and editing. **Stephan Barcikowski**: supervision, resources, funding acquisition, writing – review and editing. **Bin Cai**: validation, supervision, resources, methodology, investigation, funding acquisition, conceptualization, formal analysis, data curation, writing – review and editing.

## Conflicts of Interest

The authors declare no conflicts of interest.

## Supporting information



The authors have cited additional references within the Supporting Information [6, 9, 48, 60, 85, 86, 87, 88, 89, 90, 91, 92, 93, 94, 95, 96, 97, 98, 99, 100, 101, 102].**Supporting File**: anie73214‐sup‐0001‐SuppMat.pdf.

## Data Availability

The data that support the findings of this study are available from the corresponding author upon reasonable request.
