# Supplementary material for: Pulsed‐Laser Ablation for the Synthesis of High‐Entropy Alloy Aerogels Toward H2O2 Production and Water Decolorization
Source: Angew Chem Int Ed Engl. 2026 Jun 30;65(34):e2026722. doi: 10.1002/anie.2026722 (PMC13480746; doi:10.1002/anie.2026722)
Supplement: Supplementary file 1 [file ANIE-65-e2026722-s001.pdf]

Supporting Information

Pulsed-Laser Ablation for the Synthesis of High-Entropy Alloy Aerogels toward H_2O_2 Production and Water Decolorization

Cui Wang,[a] Varatharaja Nallathambi,[a] Lingwei Wang, Johannes Kresse, Natalia F. Shkodich, Michael Farle, René Hübner, Alexander Eychmüller, Sven Reichenberger, Stephan Barcikowski* and Bin Cai**

Dr. C. Wang, J. Kresse, Prof. A. Eychmüller
Physical Chemistry, Technische Universität Dresden, 01069 Dresden, Germany
*E-mail: alexander.eychmueller@tu-dresden.de

V. Nallathambi, Dr. S. Reichenberger, Prof. S. Barcikowski
Technical Chemistry I and Center for Nanointegration Duisburg-Essen (CENIDE), University of Duisburg-Essen, Universitaetsstr. 7, Essen, 45141 Germany
*E-mail: stephan.barcikowski@uni-due.de

V. Nallathambi
Max Planck Institute for Sustainable Materials, Max-Planck-Str.1, 40237 Düsseldorf, Germany
C. Wang, L. Wang, Prof. B. Cai
School of Chemistry and Chemical Engineering, Shandong University, 250100 Ji'nan, China
*E-mail: bin.cai@sdu.edu.cn

Dr. N.F. Shkodich, Prof. M. Farle
Faculty of Physics and Center for Nanointegration Duisburg-Essen (CENIDE), University of Duisburg-Essen, Lotharstr. 1, 47057 Duisburg, Germany

Dr. R. Hübner
Helmholtz-Zentrum Dresden-Rossendorf, Institute of Ion Beam Physics and Materials Research, Bautzner Landstrasse 400, 01328 Dresden, Germany

[a] Dr. C. Wang and V. Nallathambi contributed equally to this work.

Keywords: Laser ablation, high-entropy alloys, metal aerogels, electrosynthesis, hydrogen peroxide, water treatment

Experimental Section

Chemicals. All chemicals were used as received without further purification. Micropowders of the single metals (purchased from Chempur): Co (99.9%, ~ 74 μm), Fe (99.0%, < 60 μm), Mn (99.5%, ~ 44 μm), Ni (99.9%, < 10 μm), Cr (99+%, < 160 μm) were used as the source materials. Nafion (5 wt%), sodium borohydride ($NaBH_4$, 99.99%), methylene blue (MB), and potassium hydroxide (KOH, 85%) were purchased from Sigma-Aldrich. Rhodamine B (RhB) was purchased from Lambda Physik. Ethanol, acetone, and isopropanol are analytical reagents (AR). Milli-Q water (18.2 MΩ) was always used to prepare aqueous solutions.

Synthesis. The bulk targets of CrMnFeCoNi, CrMnCoNi, CrMnFeNi, and CrMnFeCo were prepared in 2 steps: (1) Single-*fcc* HEA micropowders (see below for XRD data) were synthesized by short-term high-energy ball milling (HEBM)[1] in a water-cooled planetary ball mill "Activator-2S" using stainless-steel cylindrical containers and balls (Ø = 7 mm). In all cases, the ball/powder weight ratio was 20:1. The vial was evacuated and then filled with Ar gas at 4 bars to prevent oxidation during the processing. The HEBM was carried out at rotation speeds of the main disk/jars: 700 rpm/1400 rpm. The milling time in Ar (dry conditions) was 90 minutes. An additional milling (10 min) in C_3H_7OH (isopropanol) was applied. (2) The HEBM micropowders were pressed under a load of 200 MPa into cylindrical targets with a diameter of 20 mm. The sintering was carried out under an argon atmosphere at 1000 °C for 24 h. The pulsed laser ablation experiments (PLAL) were carried out using a nanosecond-pulsed laser (EdgeWave GmbH) with a wavelength of 512 nm, pulse duration of 7 ns, repetition rate of 5 kHz, and pulse energy of 15 mJ. The polished bulk target was placed in a flow chamber with a continuous flow of degassed and nitrogen-bubbled liquid medium of choice (water, ethanol, or acetone) at a volume flow rate of 50 mL min^{-1}. The laser was then focused on the bulk target in a continuous circular pattern using a scanner to produce colloidal nanoparticles.

Taking the high-entropy alloy CrMnFeCoNi aerogel as an example, the typical "two-step" gelation approach is described as follows.[2]

CrMnFeCoNi aerogel. The resulting NPs solution was washed by centrifugation three times for purification. $NaBH_4$ (molar ratio of $NaBH_4$ to NPs is ~50:1) was used as the gelation agent. Thereby, the CrMnFeCoNi solvogel was synthesized after grounding for 12 h. Then, the as-prepared hydrogel was washed with water ~10 times (2~3 days), and the CrMnFeCoNi aerogel was carefully collected after supercritical CO_2 drying. A similar fabrication method was used to prepare the CrMnFeCo, CrMnFeNi, and CrMnCoNi aerogels.

Characterization. The porous structures of the as-prepared aerogels were analyzed via scanning electron microscopy (SEM) with a Hitachi FESEM SU8020 (2 kV, 10 mA). Transmission electron microscopy (TEM) images were recorded using a JEOL JEM 1400 plus equipped with a LaB_6 cathode at an accelerating voltage of 120 kV. Particle sizes were determined from TEM images by measuring 100 nanoparticles using Nano Measurer 1.2.0. High-angle annular dark-field scanning transmission electron microscopy (HAADF-STEM) imaging and spectrum imaging analysis based on energy-dispersive X-ray spectroscopy (EDS) were performed at 200 kV with a Talos F200X microscope equipped with an X-FEG electron source and a Super-X EDS detector system (FEI). Prior to STEM analysis, the specimen mounted in a high-visibility

low-background holder was placed for 2 s into a Model 1020 Plasma Cleaner (Fischione) to remove potential contamination. The phase structure and crystallinity of each gel were analyzed by powder X-ray diffraction (XRD) using a Bruker Phaser D2 diffractometer with Cu-K_α radiation ($\lambda = 1.5406$ Å). N_2 adsorption-desorption curves were obtained at standard temperature (77 K) and pressure (1 atm) with a Quantachrome Nova 3000e. Before the measurements, the samples were outgassed at 323 K for ~24 h under vacuum. The value of pore volume and BET surface area are based on the average from three-time measurements. X-ray photoelectron spectroscopy (XPS) of the aerogels was conducted using a VersaProbe IITM from Ulvac-Phi employing the Al-Kα line at 1486.6 eV and a spot size of 100 µm at an energy resolution of 0.5 eV. A hemispherical analyzer (with an angle of 45° between the sample surface and the analyzer) and dual-beam charge neutralization were used, whereby the detector was operated at a pass energy of 23 eV. The CasaXPS software was used to perform the peak deconvolution with a Shirley[3] background applied to the individual peak fits. The acquired high-resolution XPS spectra were calibrated according to the binding energy of adventitious carbon (284.8 eV) determined during deconvolution of the C 1s spectrum of each sample.[4] As transition metal oxides such as Fe, Co, Ni, Cr, and Mn tend to show complex peak shapes e.g. due to satellite signals (Shake-up/off, plasmons or Auger electrons) a single Gauss-Lorenzian or asymmetric Lorenzian is not sufficient to adequately describe the peak shape of e.g. FeO, Fe_2O_3 or FeOOH via fitting a Fe^{2+} and Fe^{3+} feature for all possible oxides. In general, asymmetric Lorentzians can be used for the elementary metal peaks. In turn, the analysis of the transition metal oxide and hydroxide signals in the Fe2p, Co2p, Ni2p, Cr2p, and Mn2p ranges had to be performed using a cumulative combination of several Gauss-Lorenzian. Hereby, the complex peak shape of each transition metal oxide (e.g. FeO, Fe_2O_3, FeOOH or CoO, $Co(OH)_2$ or CoOOH etc.) is described by the sum of typically up to 4-6 sub-peaks. These sub-peaks (typically a 50:50 Gauss-Lorenzian for each sub-peak) are constrained in such a way, that the relative position (peak shift) as well as the area ratio of all peaks and their respective peak width are fixed in a set of constraints. Consequently, if one would want to shift the first sub-peak by 1 eV also all other sub-peaks that correspond to the same transition metal oxide are shifted by 1 eV, accordingly. In the same way, if the area of the first sub-peak is increased by a factor of e.g. 2, so are the areas of all sub-peaks that correspond to the same oxide, respectively. The peak width of all sub-peaks in turn, is mostly fixed and only allowed to vary by ±0.2 eV to account for any peak broadening or narrowing that occurred in our measurement compared to the measurement of the reference spectrum taken from literature. All the constraints used during the peak fitting of the $2p_{3/2}$ spectra of all elements can be found in **Table S5**. Additionally, we kindly refer the reader to the guideline of M.C. Biesinger in *X-ray Photoelectron Spectroscopy (XPS) Reference Page*s (*https://www.xpsfitting.com*). During the analysis, also the Auger contributions from Fe, Co, and Ni were considered during peak deconvolution. Yet, the Auger contributions had to be approximated by Gauss-Lorenzian where the peak position was constrained to the respective Auger contribution positions with an allowed variation of ±2 eV. As an example, in case of the Fe2p signal analysis two Auger signals stemming from CoO (constrained to 711.8-715.8 eV, and 715-719 eV) as well as NiO (constrained to 712.4-712.6 eV) had to be considered.[5] The assumption of two CoO Auger signals was taken from own

experimental data of a Co_3O_4/CoO mixed oxides where the ratio of both signals appeared to also correlate with the Co_3O_4 and CoO ratio (but was not further discussed here). The peak areas calculated after deconvolutions of the 2p3/2 signals were used for surface composition quantification. All K-edge absorption spectra (XAS) measurements in this work were performed at the BL 16U1 beamline of the Shanghai Synchrotron Radiation Facility.[6] Ex situ spectra were recorded for 10 min at the K-edge in the transmission and fluorescence mode for all elements. Data processing for energy calibration, interpolation, normalization and averaging was achieved using the Demeter system.[7] Given the limited signal-to-noise ratio and the multi-component merit of the as-prepared aerogels, we restrict the EXAFS discussion to qualitative or semiquantitative trends derived from consistently processed k-space and R-space data (fixed k-range and window function). No coordination numbers or bond lengths are claimed without model-dependent fitting. ICP-MS analysis was carried out using a NexION 350X (PerkinElmer), where the solutions were prepared by digesting the catalysts in aqua regia for 24 hours. The Raman spectra was measured on a LabRAM HR Raman microscope with a 532 nm laser. Fourier Transform Infrared Spectroscopy (FTIR) analysis was performed using Bruker Invenio R instrument, accompanied with OPUS 8 software for air-referenced baseline correction and normalization. Ultraviolet-visible (UV-Vis) absorption spectra were recorded on a Cary 60 UV-Vis spectrophotometer. The supercritical drying was performed in an autoclave type 13200J0AB from Spi Supplies.

Density functional theory calculations. First-principle's calculations based on density functional theory (DFT) were performed using the CP2K package, which employs a mixed Gaussian and plane-wave scheme (see *CP2K Open Source Molecular Dynamics (https://www.cp2k.org/quickstep?utm_source)*). The exchange-correlation interactions were described by the generalized gradient approximation with the Perdew-Burke-Ernzerhof (PBE) functional[8], and core-valence interactions were treated using Goedecker-Teter-Hutter (GTH) pseudopotentials[9]. DZVP-MOLOPT-SR-GTH Gaussian basis sets were used for all atoms. Long-range dispersion interactions were considered using the DFT-D3 method with Becke-Johnson damping.[10] To represent the surface of the multimetallic aerogel, a periodic slab model was constructed based on the fcc surface, which is commonly observed for the alloy phase. The slab consisted of four atomic layers with a 4 × 4 surface supercell. A vacuum layer of 15 Å was introduced along the surface-normal direction to avoid artificial interactions between periodic images. The bottom two atomic layers were fixed to mimic the bulk-like region, while the upper two atomic layers and adsorbates were fully relaxed. The Cr, Mn, Fe, Co, and Ni atoms were arranged in the slab to approximate the local chemical environment of the multicomponent alloy. Specifically, the five elements were distributed nearly equimolarly in the surface and subsurface layers, and several possible atomic arrangements were examined, and the most stable configuration was used for adsorption calculations. The adsorption sites considered in this work included top sites involving different neighboring metal atoms. The reported adsorption energies were obtained from the most stable optimized adsorption configurations. An auxiliary plane-wave cutoff of 400 Ry (relative cutoff of 55Ry) was used. Self-consistent-field (SCF) convergence was aided by Fermi-Dirac smearing at 300 K, with an electronic energy convergence criterion of 1.0×10^{-5} Hartree. Geometry optimizations were

performed using the BFGS algorithm until the maximum and RMS forces were below 4.5×10^{-4} and 3.0×10^{-4} Hartree Bohr^{-1}, respectively, and the maximum and RMS atomic displacements were below 3.0×10^{-3} and 1.5×10^{-3} Bohr, respectively. Three-dimensional periodic boundary conditions were applied throughout the entire calculation process. It should be noted that the present DFT model is a simplified representation of the CrMnFeCoNi HEA aerogel surface. The real aerogel may contain structural disorder, defects, grain boundaries, surface oxidation/hydroxylation, and electrolyte-induced reconstruction, which are not fully captured in the idealized slab model. Therefore, the DFT results are mainly used to provide qualitative mechanistic insight and compare relative trends in intermediate adsorption rather than to quantitatively reproduce the complete catalytic interface under operating conditions.

Electrochemical tests. *$2e^-$ ORR performance.* The performance of the selective oxygen reduction reaction was obtained in a three-electrode cell via an Autolab potentiostat system (PGSTAT 128N, Metrohm Autolab B.V., The Netherlands) with a rotating ring-disc electrode (RRDE) setup at room temperature. In particular, a glassy carbon (GC) disc (geometric area of 0.196 cm^2) with Pt ring electrode (series No. AFE6R1PT, Pine Research Instrument, theoretical collection efficiency (i.e. vendor value) of 25%), an Ag/AgCl electrode (Pine Research Instrument, KCl aqueous solution of 4 M), and a Pt foil were employed as the working, reference, and counter electrode, respectively. The practical collection efficiency (denoted as *N*) was calibrated in an electrolyte (150 mL) of Na_2SO_4 (0.5 M) and $K_3Fe(CN)_6$ (0.05 M) at 1600 rpm.[11] A potential of 1.2 V_{RHE} was applied on the Pt ring to monitor the generated H_2O_2 on the disk electrode.[12] The measured value of N was 20.6% ($N\% = -100 \times \frac{I_r}{I_d}$, **Figure S20**), which is used for the further calculation of the H_2O_2 selectivity. All the reported potentials are referred to the reversible hydrogen electrode (RHE) scale ($E_{RHE} = E_{Ag/AgCl} + 0.0591 \times pH + 0.197$). The applied potential of the ring was 1.2 V_{RHE} to capture the generated H_2O_2.

For the catalyst ink, ~0.5 mg catalyst, 419 µL isopropanol, and 17.6 µL Nafion (0.5% in ethanol) were mixed and then sonicated for 30 min in an ice-water bath. Afterward, 2.6 µL catalyst ink was carefully dropped onto the GC disk of the RRDE electrode, followed by drying at room temperature (mass loading of 3.793 $\mu g\ cm_{disk}^{-2}$). Before the activity test, both the GC disc with the catalyst and the ring electrode were cleaned by cyclic voltammetry (CV) curves ranging from 0.1 to 0.67 V at 100 mV/s in N_2-saturated KOH aqueous solution (0.1 M). Linear sweep voltammetry (LSV) curves were recorded with a scan rate of 20 mV s^{-1} and a rotating speed of 1600 rpm in O_2-saturated KOH aqueous solution (0.1 M). For the stability test, CV curves were employed in the range of 0.1 to 0.67 V at 100 mV s^{-1} in an O_2-saturated KOH aqueous solution (0.1 M).

Background subtraction was applied during data processing, and both the ring current and the disk current were obtained by subtracting the N_2-saturated current density from the O_2-saturated value,[13] as far as possible avoiding any capacitive contributions and any residual background oxidation or reduction at the ring and disk.[14] The selectivity of H_2O_2 (H_2O_2 selectivity %), the number of transferred electrons (n) and the Faradic Efficiency (FE%) during oxygen reduction reaction were calculated using the following equations,[11,15] respectively:

$$H_2O_2 \text{ selectivity\%} = \frac{200 \times I_r/N}{I_d + I_r/N}$$

$$n = \frac{4 \times I_d}{I_d + I_r/N}$$

$$FE\% = 100 \times \frac{I_r/N}{I_d}$$

wherein I_r and I_d are the ring and disc currents, while N is the collection efficiency (practical collection efficiency of 20%[11]).

H_2O_2 production. An H-cell with Nafion 117 membrane (Sigma-Aldrich) separator was used for the bulk production of H_2O_2 by a chronoamperometry method (**Figure 4a** in the main text). Each compartment was filled with 40 mL O_2-saturated 0.1-M KOH aqueous solution. The effect of applied potentials was investigated as follows. A piece of carbon cloth (Fuel cell store, 1 cm x 1.5 cm) deposited with CrMnFeCoNi ink of 3.9 μL and an Ag/AgCl electrode in the cathode compartment served as working and reference electrodes, respectively. A Pt foil was set in the anode compartment. During the measurement, the electrolyte solution was magnetically stirred at 400 rpm. A titration with $KMnO_4$ solution (1 mM) was taken to calculate the produced H_2O_2 amount using the equation: $2MnO_4^- + 5H_2O_2 + 6H^+ = 2Mn^{2+} + 5O_2 + 8H_2O$.[11] The details are as follows. After electrocatalysis at different applied potentials (0.1 V_{RHE}, 0.15 V_{RHE}, 0.2 V_{RHE}, 0.25 V_{RHE}, 0.3 V_{RHE}) for 60 min, 10.0 mL of the electrolyte from the cathode compartment was collected and mixed with 10 μL of H_2SO_4 aqueous solution (0.1 M), followed by the $KMnO_4$ titration. Once the light pinkish color appeared for 30 s, the endpoint of the titration was reached. Each titration was performed 3 times and the average value is reported.

Determination of the electrochemically active surface area (ECSA). The ECSA of the resulting aerogels was estimated by determining the double-layer capacitance (C_{dl}) of the system from the corresponding CV curve.[16] For each electrode, CV curves were recorded at different scan rates (20, 40, 60, 80, 100 mV s^{-1}) in the potential window of 0.886 V_{RHE} -0.986 V_{RHE}, in which no Faradaic process occurs. Plotting the current density as a function of the scan rate yields a straight line with a slope corresponding to C_{dl}. Then, the ECSA value of each catalyst was estimated using the following equation: $ECSA = C_{dl}/C_s$, where C_s is the general specific capacitance of 0.04 mF cm^{-2} based on a typical value reported for metal electrodes in aqueous KOH solution.

Water treatment process. The pollutant decolorization was performed in a vial with different substrates, and the concentration of the pollutants (MB or RhB) was fixed at 16 mg L^{-1}. The solution of MB or RhB was freshly prepared. The 0.1 M KOH electrolyte from the H-type cell was directly used as an H_2O_2 donor. The mixture recipe of i, ii, iii and iv in **Figure S25** are: (i) 1 mL MB or RhB (16 mg L^{-1}), (ii) 0.5 mL MB or RhB (32 mg L^{-1}) + 0.5 mL electrolyte, and 0.5 mL MB or RhB (32 mg L^{-1}) + 0.5 mL electrolyte + 0.5 mg CrMnFeCoNi HEA aerogel in (iii) dark and (iv) natural light condition. The concentration of various solutions was evaluated by UV-vis spectroscopy (Cary 60).

Figures

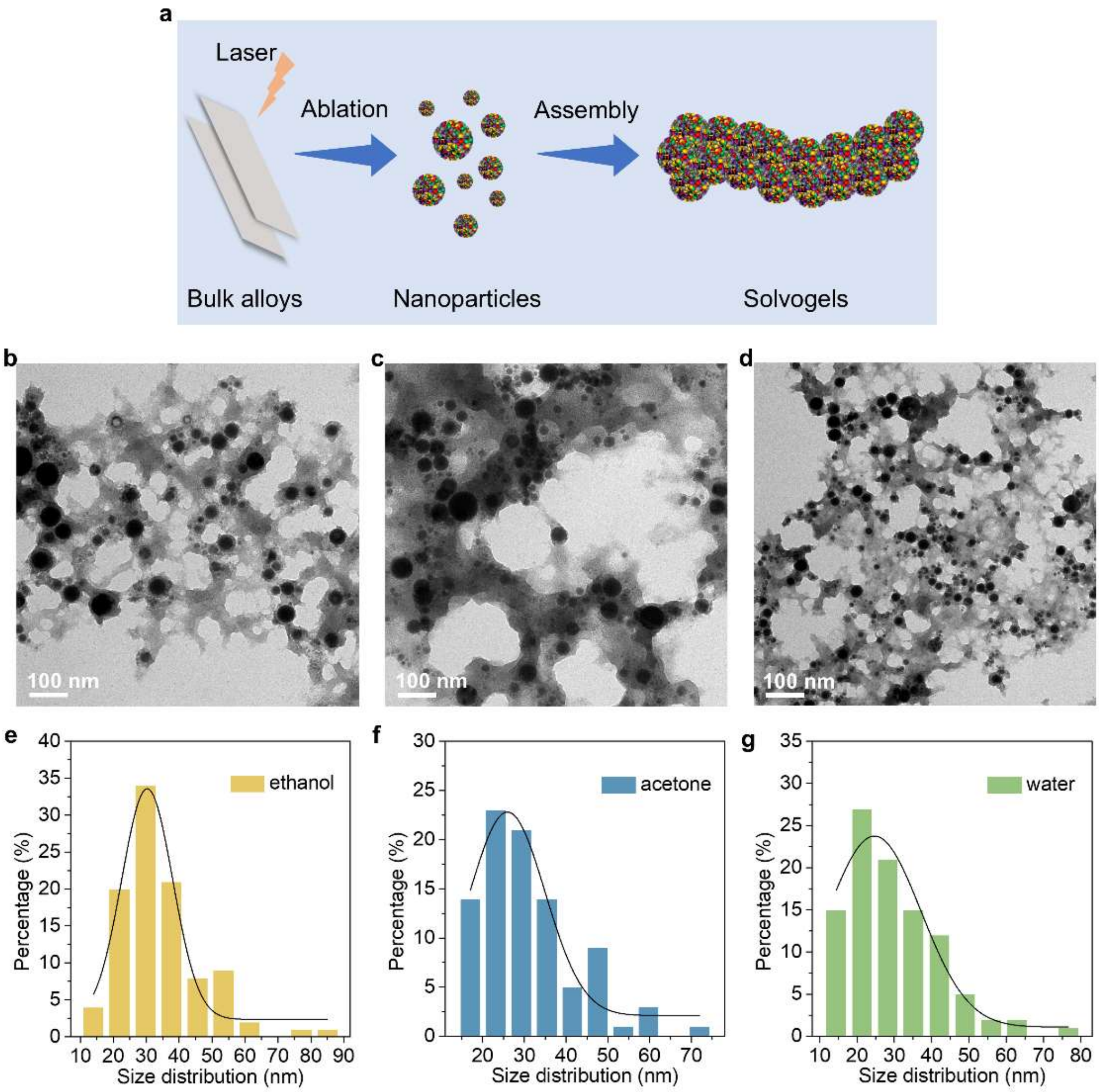

Figure S1 (a) Scheme of pulsed-laser ablation for the synthesis of HEA aerogels via a typical "two-step" approach. (b-d) TEM images and (e-g) histograms of the corresponding size distribution of the resulting HEA (CrMnFeCoNi) nanoparticles (NPs), obtained from laser ablation in (b and e) ethanol, (c and f) acetone, and (d and g) water. The diameters of more than 100 NPs in (b-g) were measured by Nano Measurer 1.2.0.

NPs resulting from the laser ablation in ethanol and acetone were found to be capped by carbon shells resulting from solvent disintegration during laser irradiation.[17] All samples show a broad size distribution in the range of 14–20 nm, depending on the reaction solvent, i.e. are 20 nm for ethanol, 17 nm for acetone, and 14 nm for water.

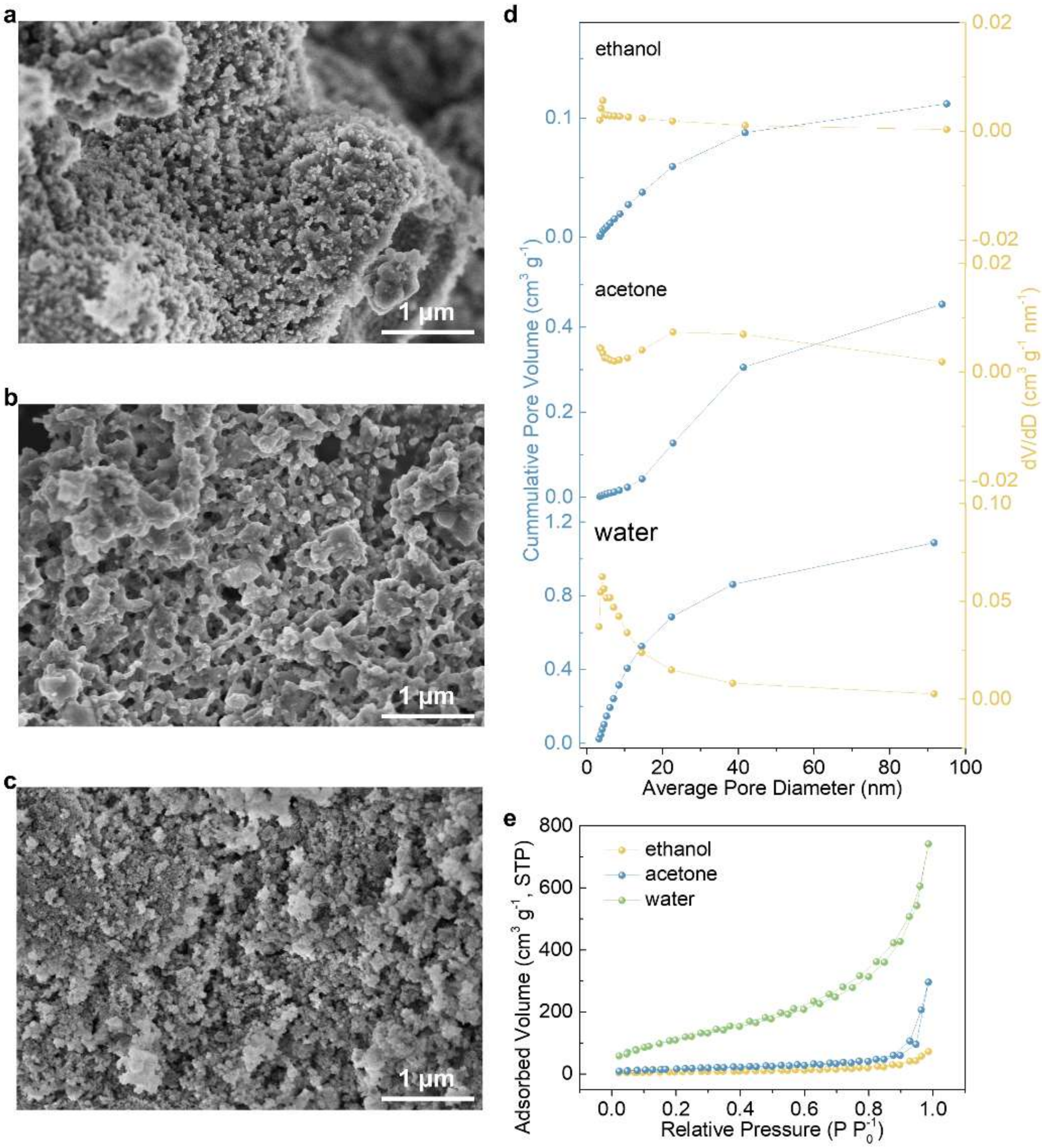

Figure S2 (a-c) SEM images, (d) the corresponding cumulative pore volumes (left) and pore size distributions (right), and (e) N_2 adsorption-desorption isotherms of the CrMnFeCoNi HEA aerogels, where the building blocks were synthesized in (a) ethanol, (b) acetone, and (c) water.

During SEM characterization, it was found that the electron beam was difficult to focus on the surface of the CrMnFeCoNi HEA aerogel synthesized in water compared to those in ethanol and acetone, due to charging effects, suggesting that more metal oxides were fabricated in the water-related synthetic system. All these HEA aerogels exhibit completely different morphologies compared to typical metal aerogels,[18] thus, it is difficult to identify parameters in terms of ligaments. The building blocks in the HEA aerogels synthesized in acetone are relatively thicker than others (**Figure S2b**), which contributes to a larger pore volume (**Table S1**) in this case. More importantly, the overall properties, such as pore size or volume, and the BET surface area, can be evidenced for the formation of aerogels rather than bulk-like materials.

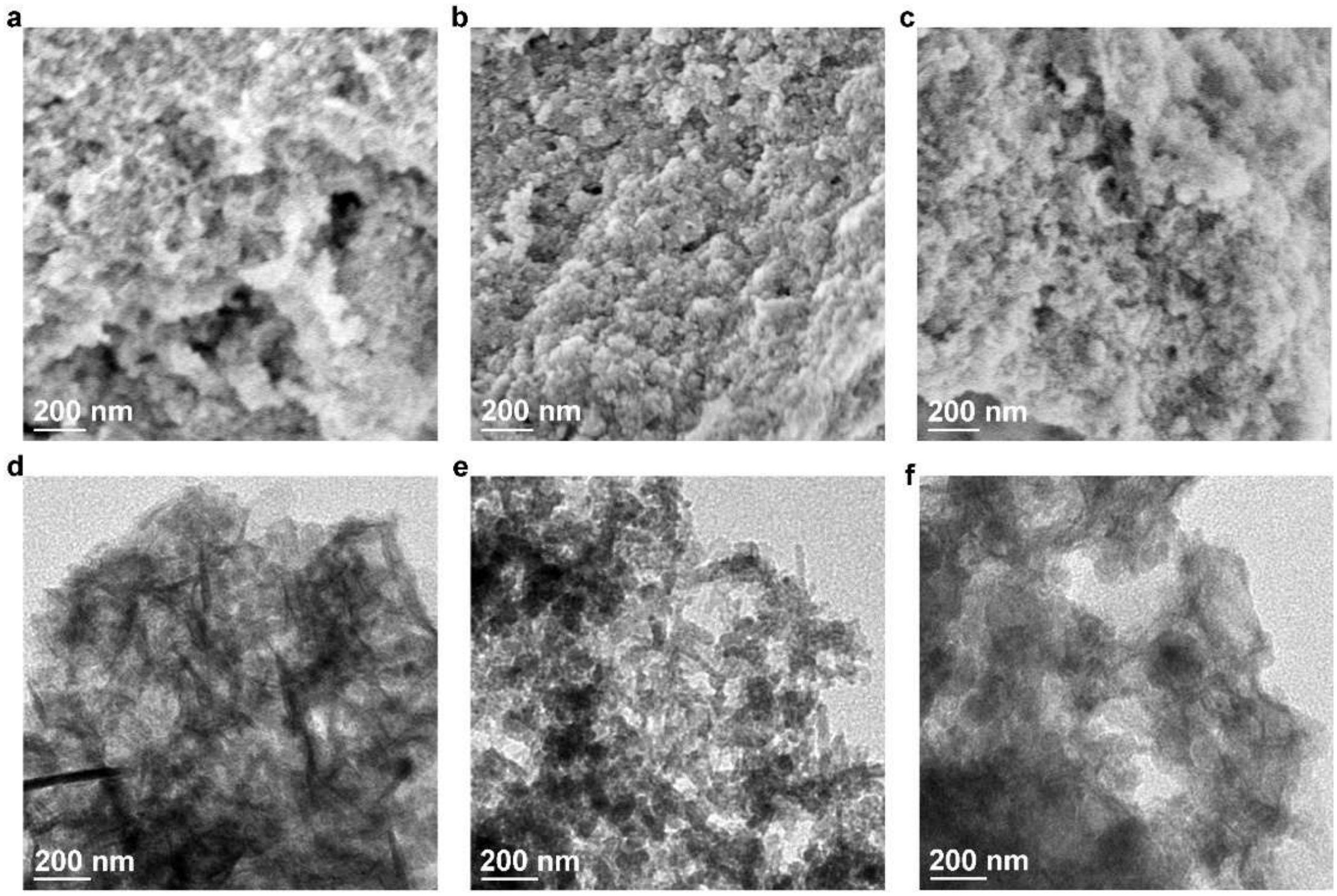

Figure S3 SEM (a-c) and TEM (d-f) images of the as-prepared CrMnCoNi (a and d), CrMnFeNi (b and e), and CrMnFeCo (c and f) aerogels.

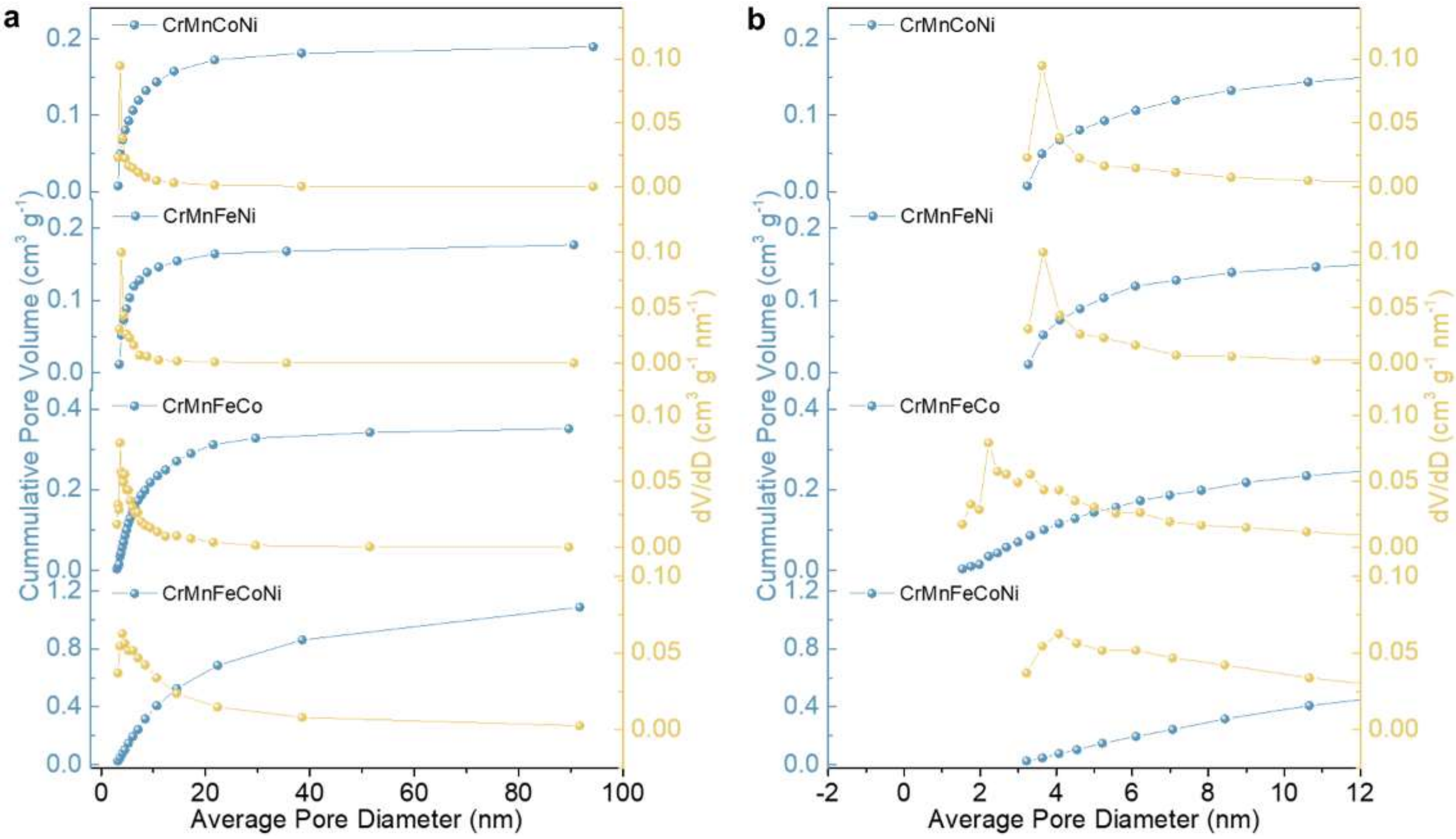

Figure S4 Cumulative pore volumes (blue) and pore size distributions (yellow) of the indicated MEA and HEA aerogels. Panel (b) is the local magnification of panel (a).

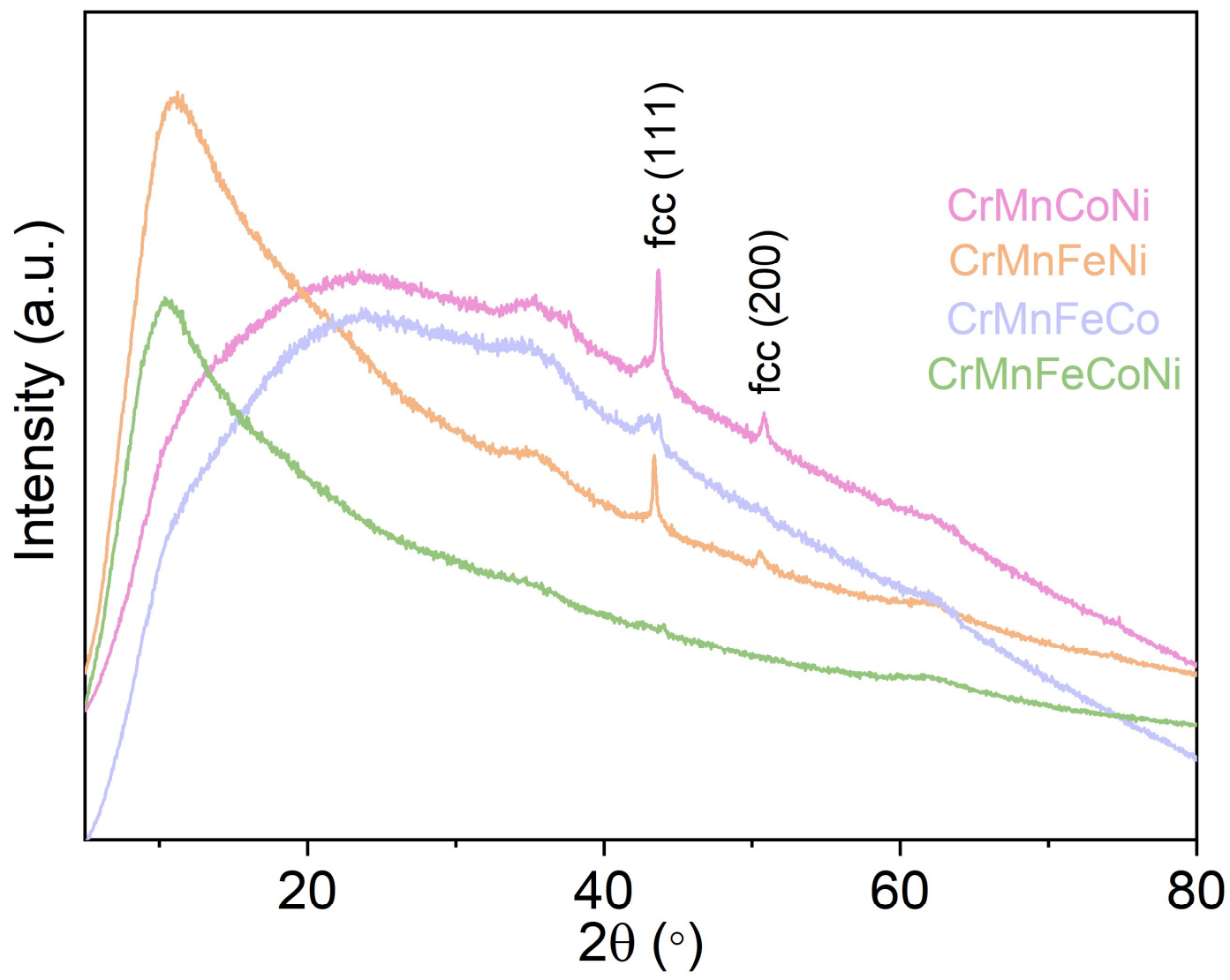

Figure S5 XRD patterns of the as-prepared HEA and MEA aerogels with different compositions from laser-fabricated nanoparticles in water.

The resulting aerogels with different compositions were synthesized in water by assembling the corresponding nanoparticles, followed by supercritical CO_2 drying. It is observed that broad diffuse diffraction reflections are present at 2θ ranges of 5-30° in the resulting aerogels, indicating the presence of an amorphous phase contribution. Additionally, the peaks at around 42° and 50° represent the formation of an fcc phase in the resulting aerogels.[19]

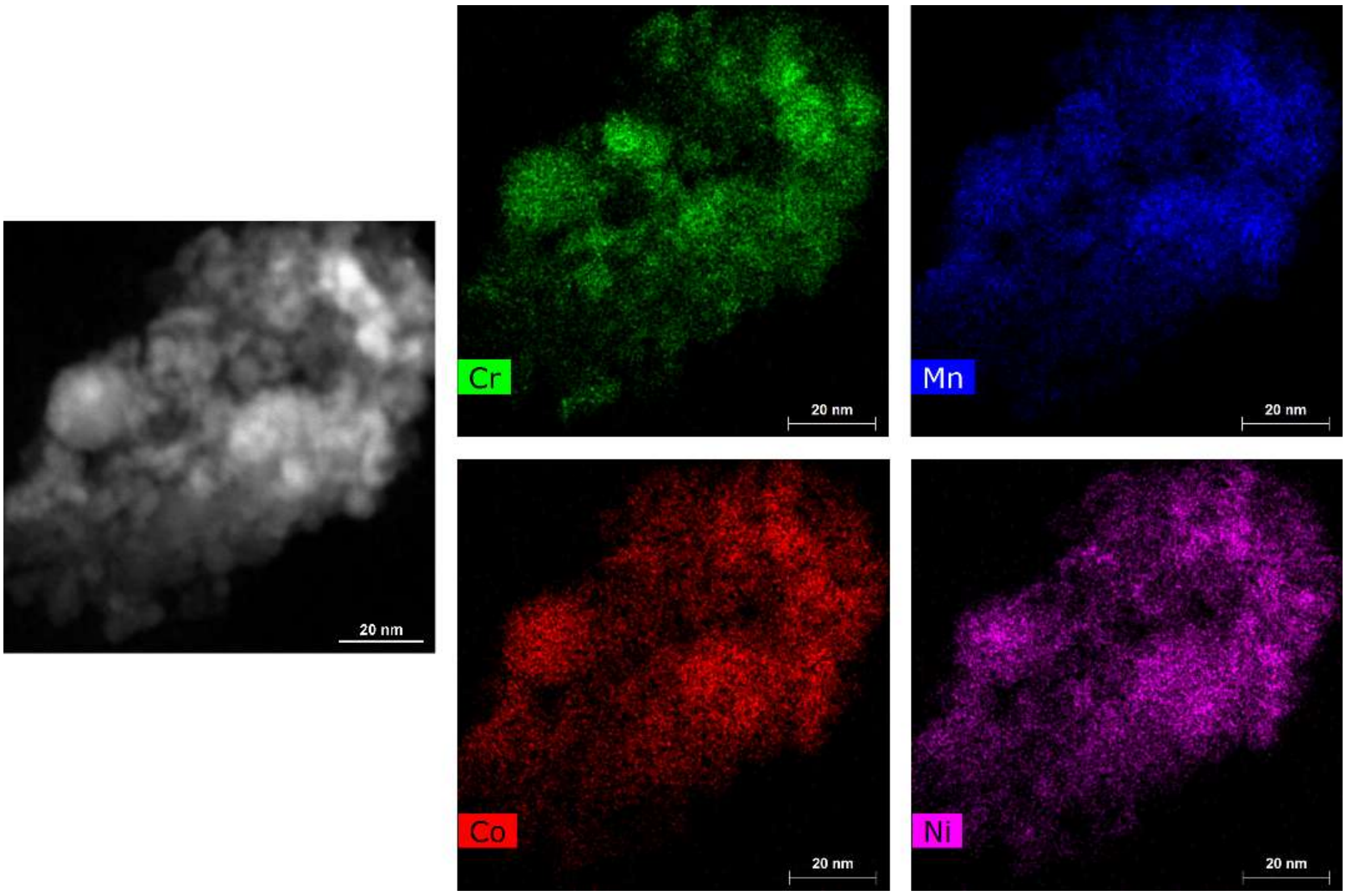

Figure S6 HAADF-STEM image and corresponding EDS-based element distribution maps of Cr, Mn, Co, and Ni in the as-prepared CrMnCoNi aerogel.

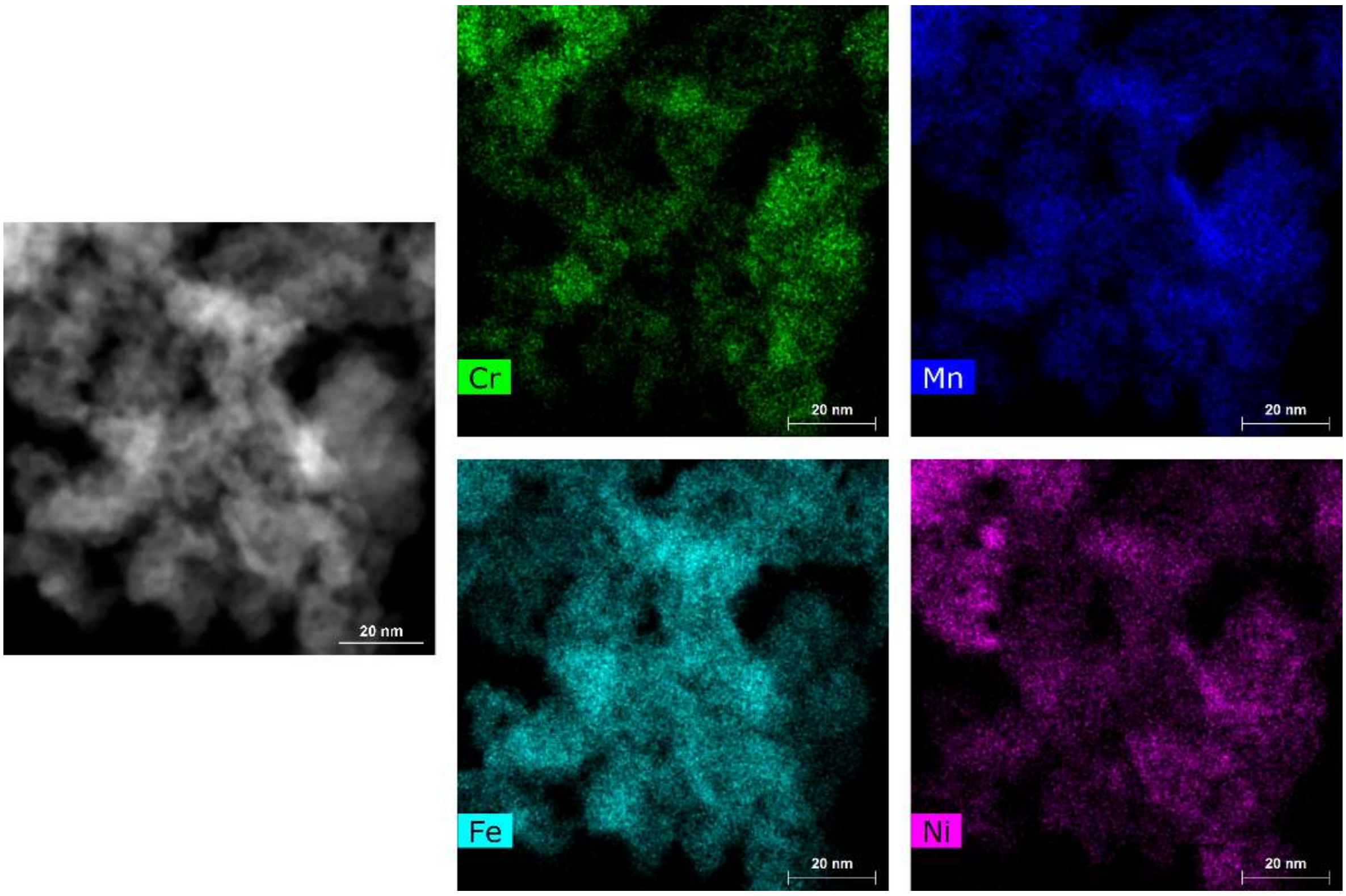

Figure S7 HAADF-STEM image and corresponding EDS-based element distribution maps of Cr, Mn, Fe, and Ni in the as-prepared CrMnFeNi aerogel.

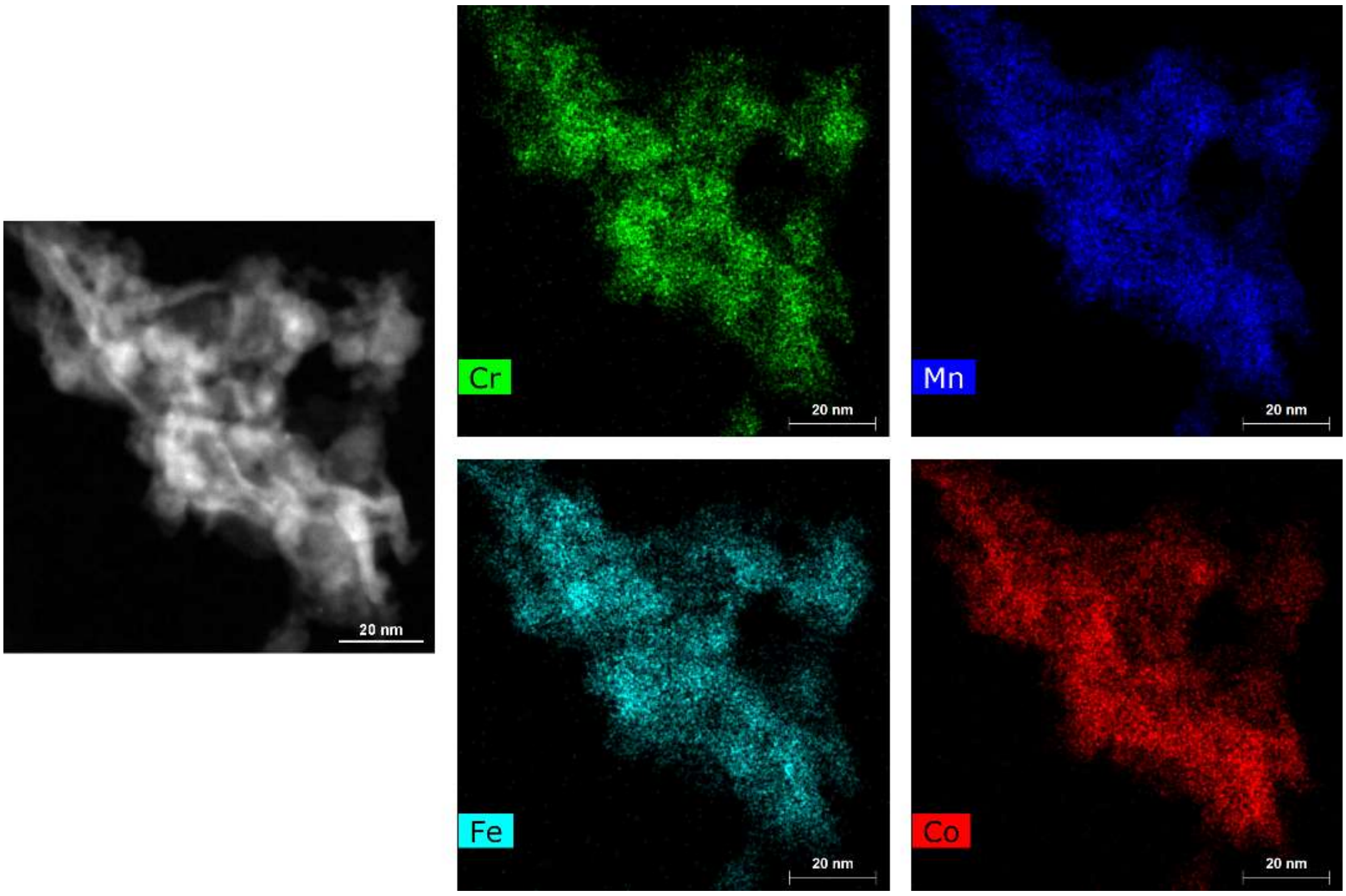

Figure S8 HAADF-STEM image and corresponding EDS-based element distribution maps of Cr, Mn, Fe, and Co in the as-prepared CrMnFeCo aerogel.

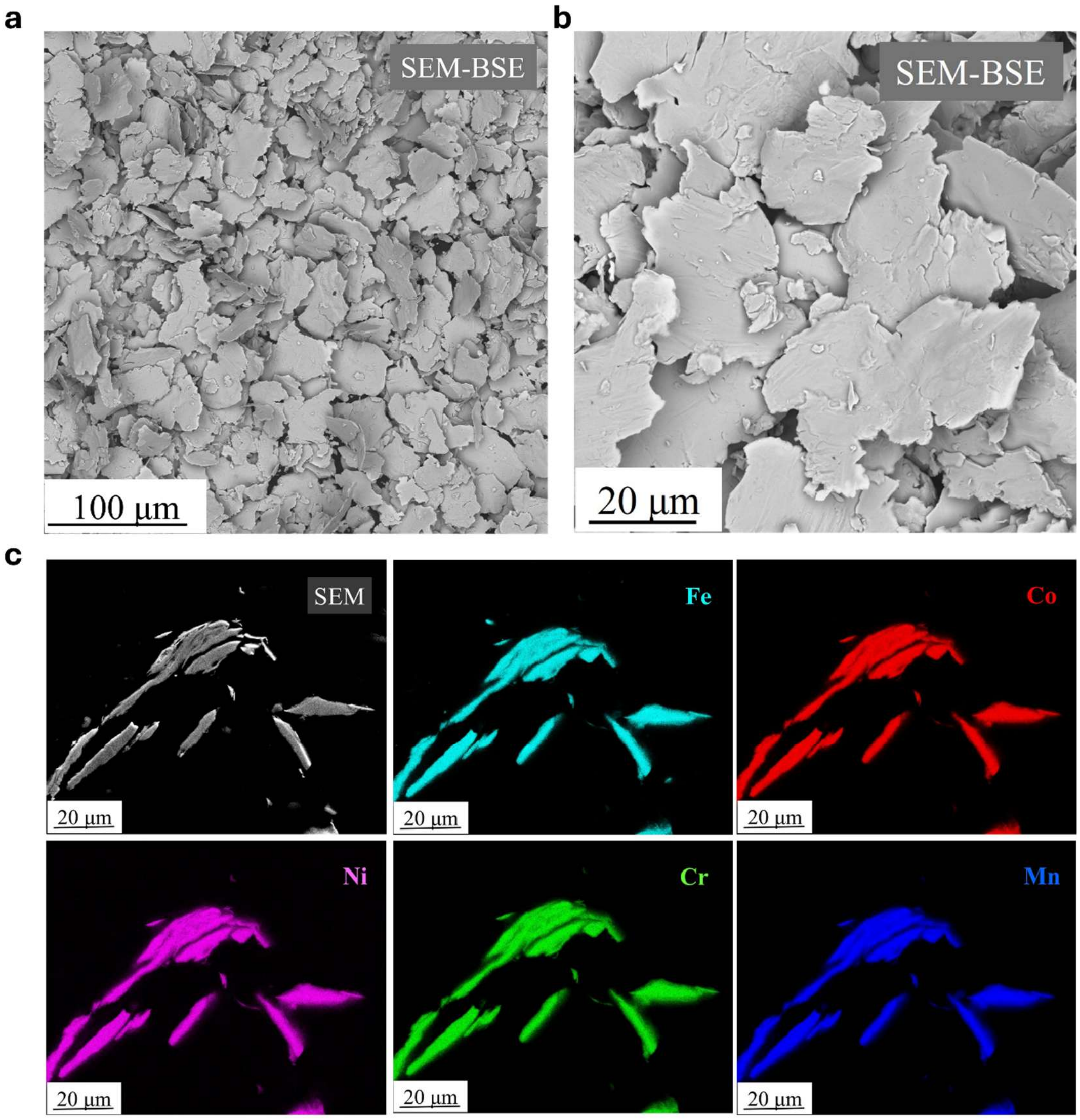

Figure S9 Back-scattered (BSE) SEM images (a, b) and SEM-EDS (c) of the CrMnFeCoNi ball-milled micropowders.

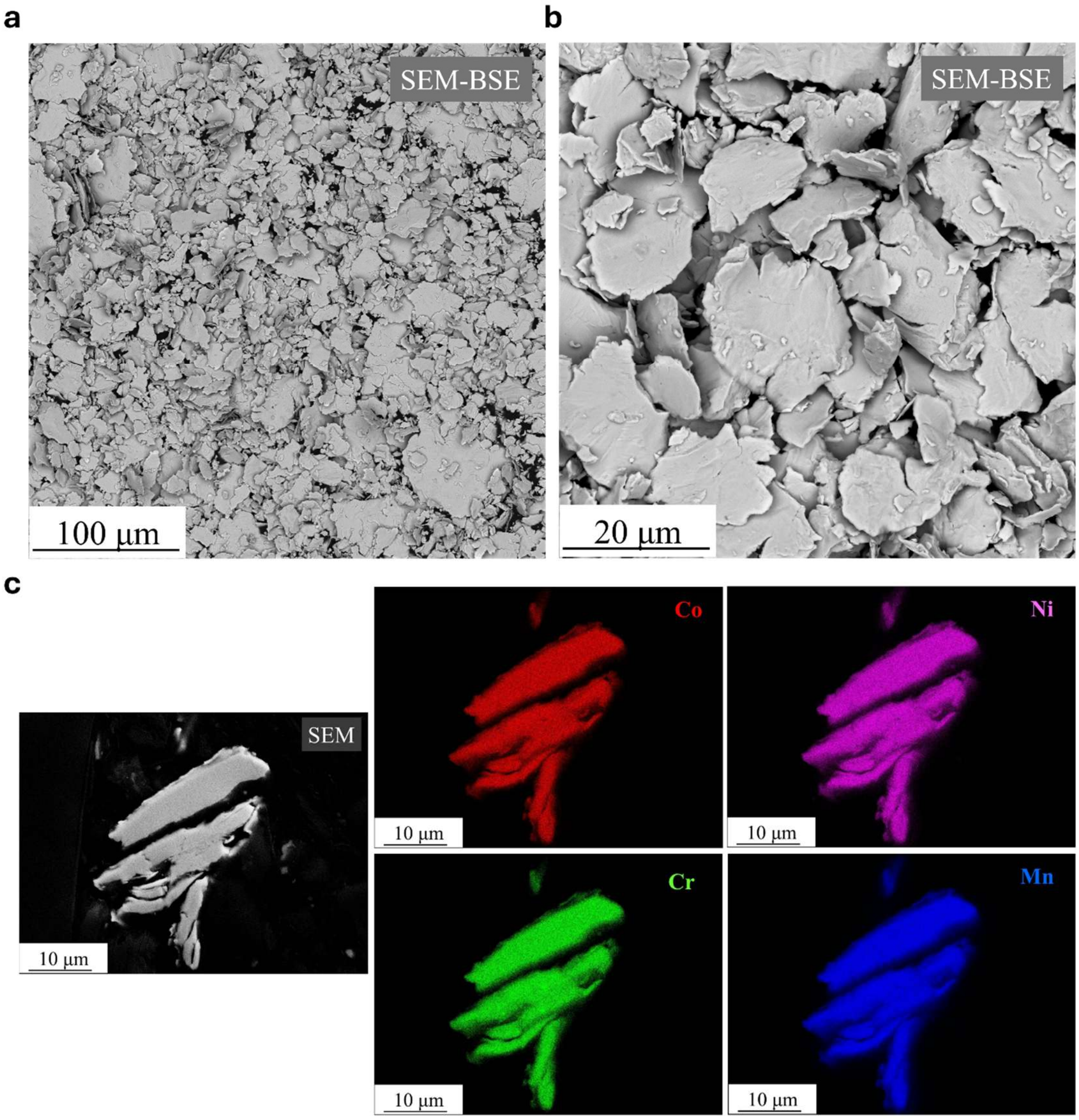

Figure S10 Back-scattered (BSE) SEM images (a, b) and SEM-EDS (c) of the CrMnCoNi ball-milled micropowders.

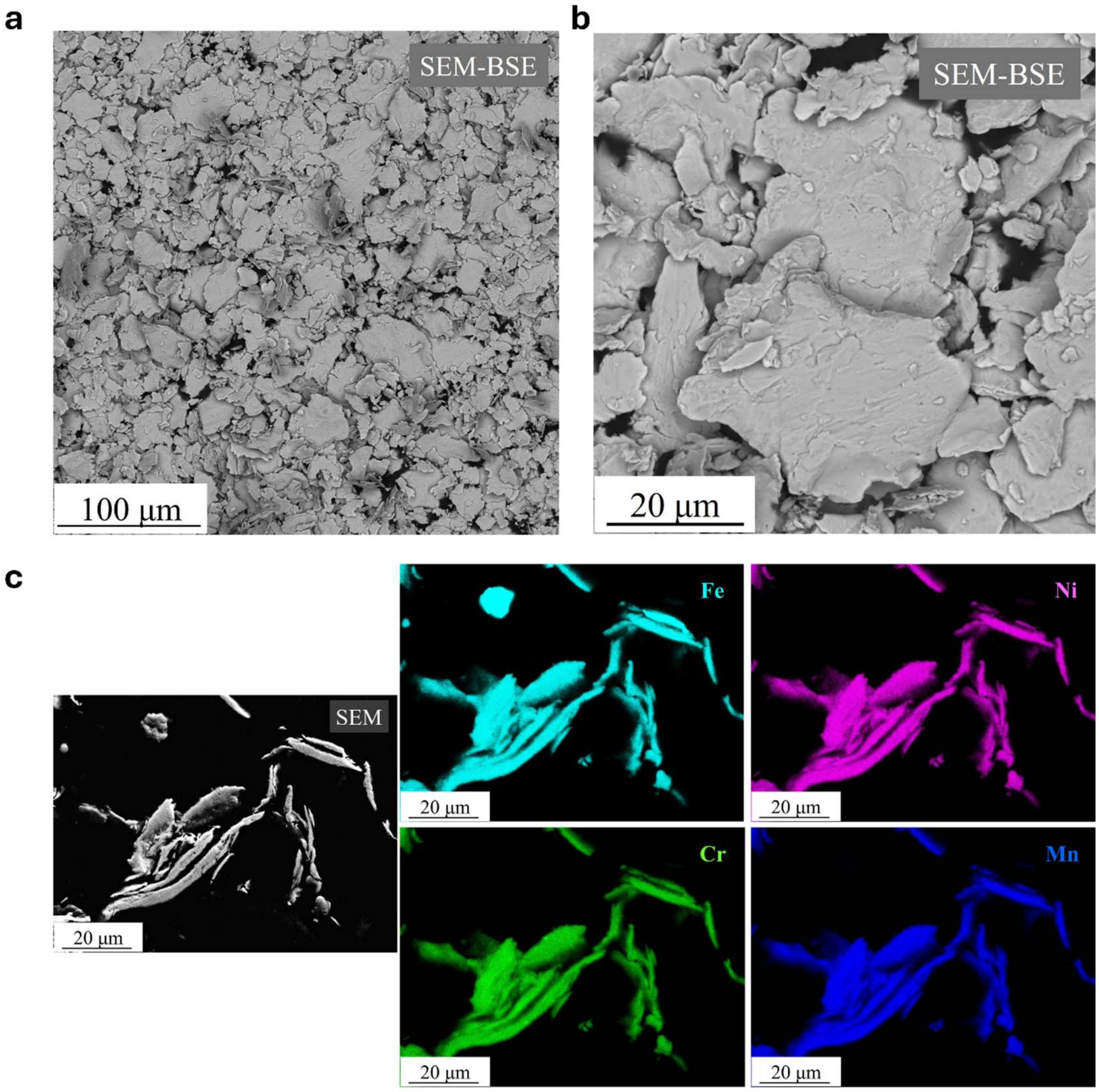

Figure S11 Back-scattered (BSE) SEM images (a, b) and SEM-EDS (c) of the CrMnFeNi ball-milled micropowders.

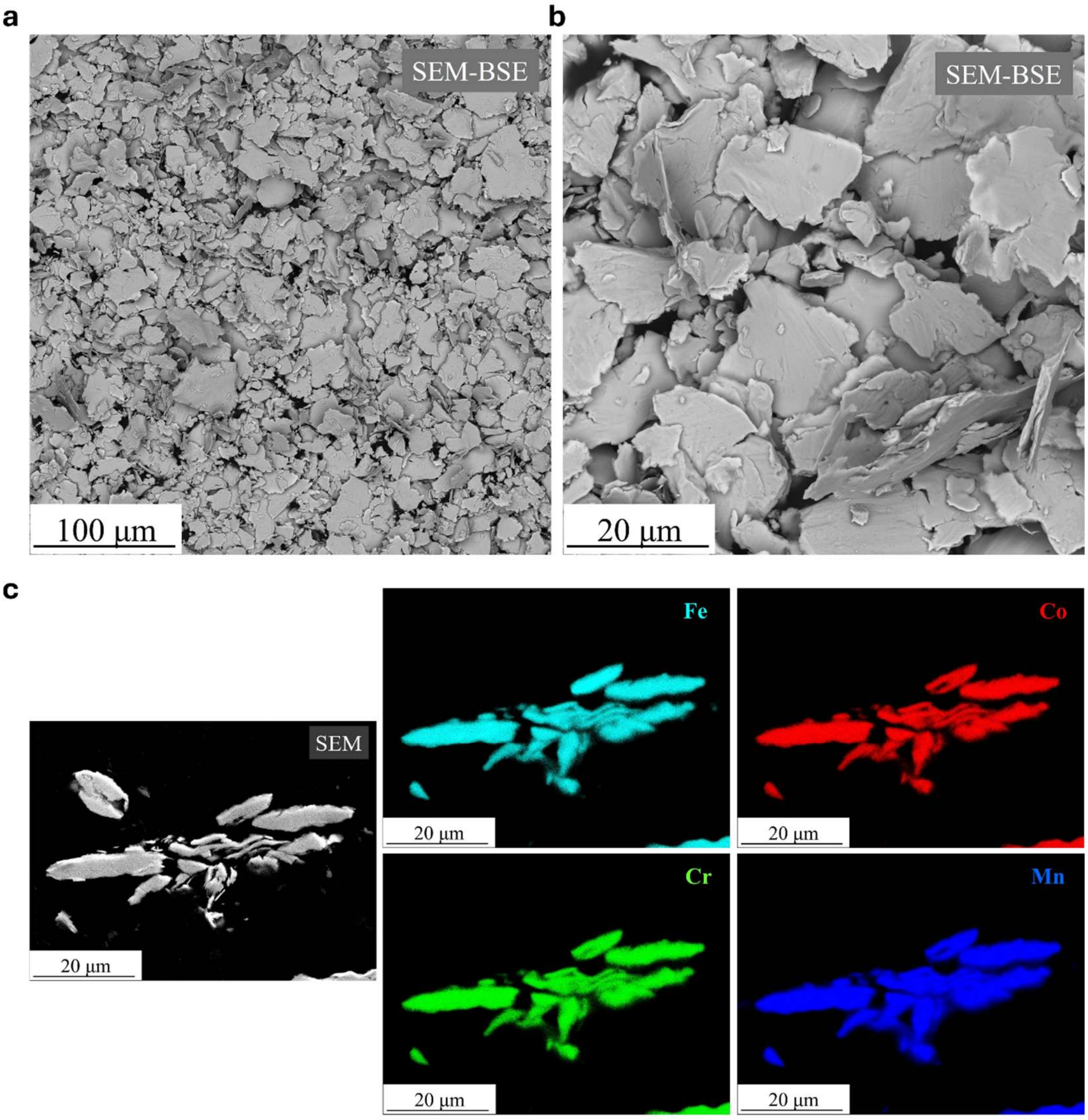

Figure S12 Back-scattered (BSE) SEM images (a, b) and SEM-EDS(c) of the CrMnFeCo ball-milled micropowders.

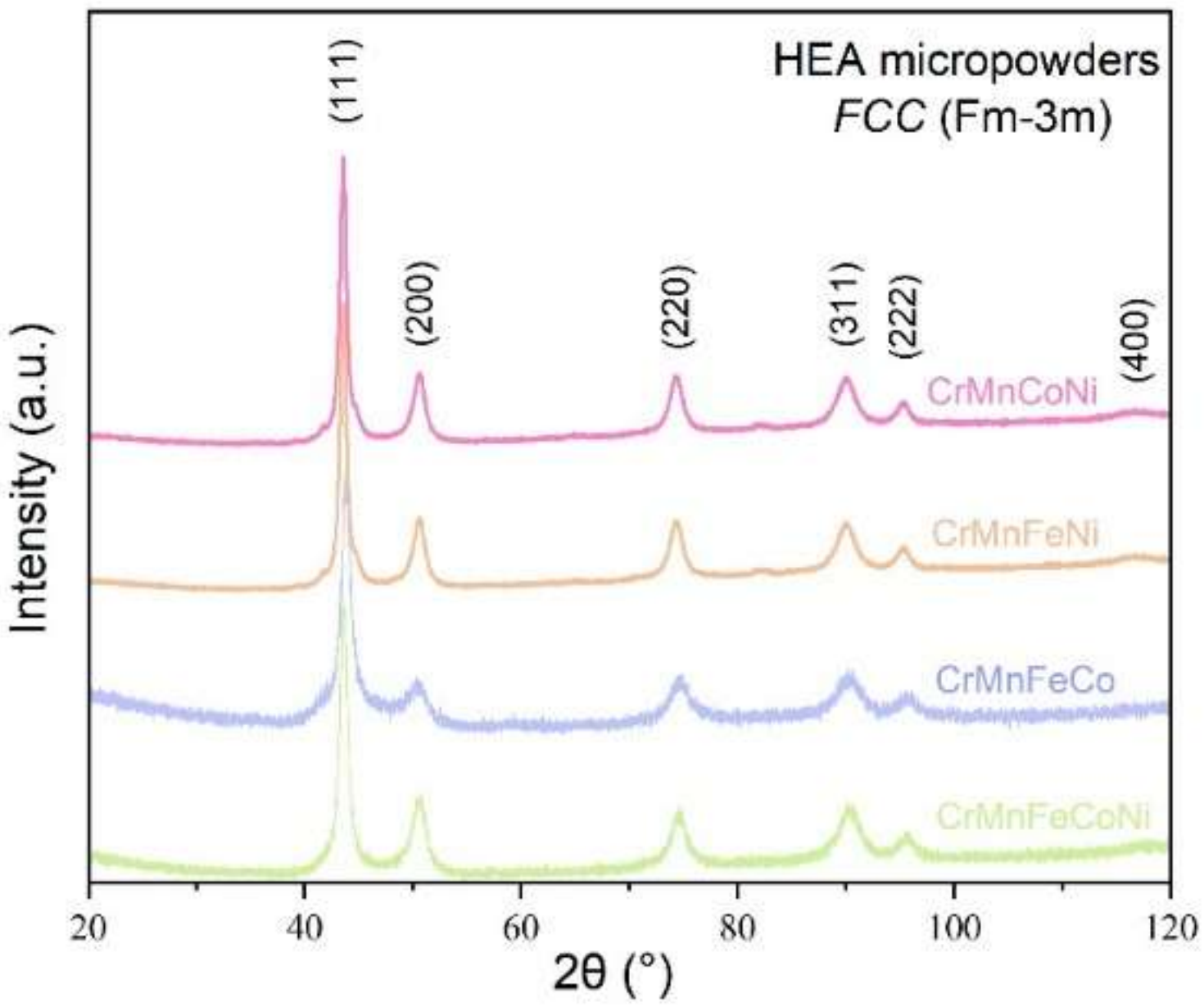

Figure S13 XRD patterns (normalized) of CrMnFeCoNi, CrMnFeCo, CrMnFeNi, CrMnCoNi ball-milled HEA micropowders (90+20* min at 700/1400 rpm). *milling time in C_3H_7OH.

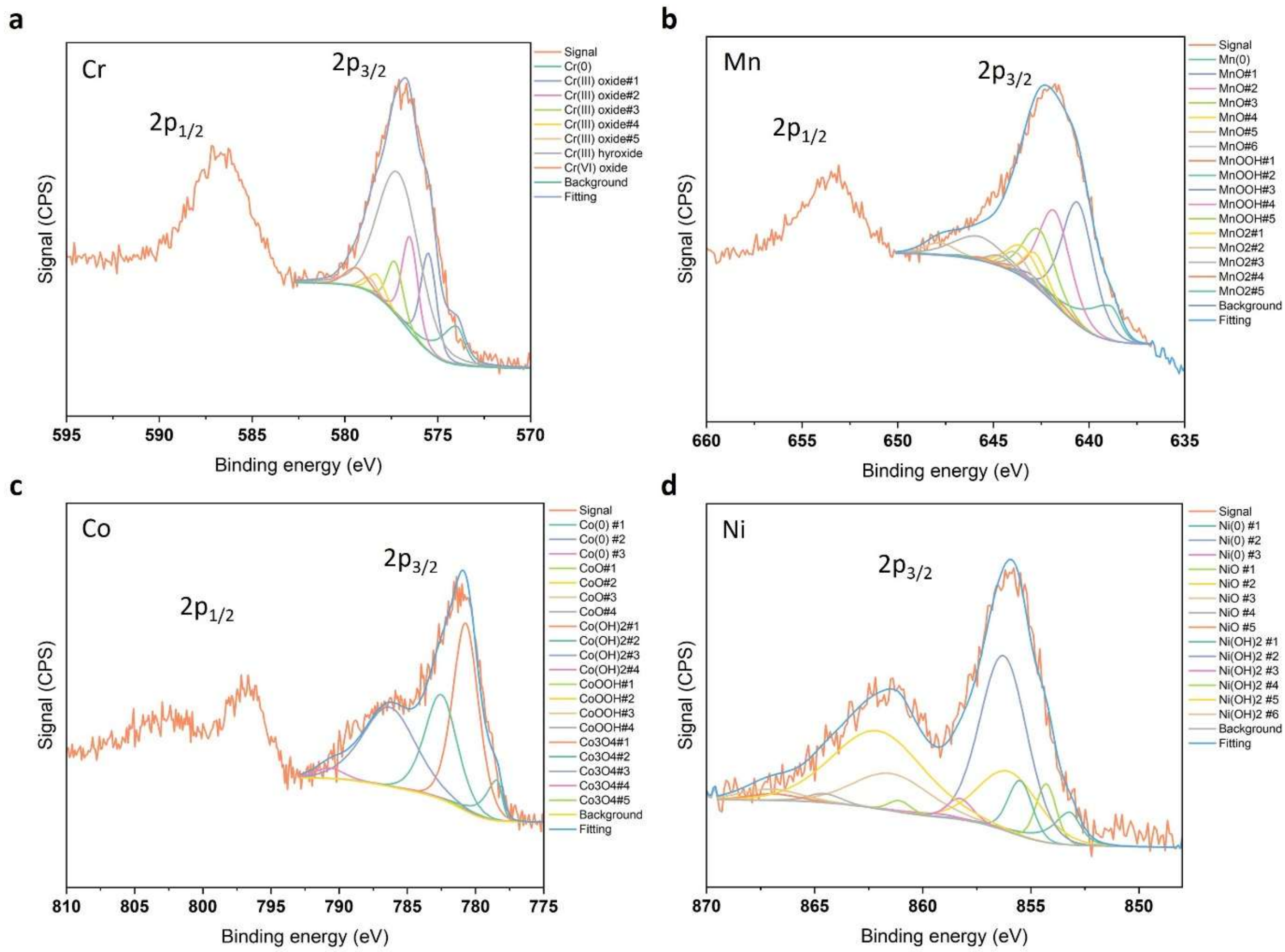

Figure S14 Deconvoluted Cr 2p (a), Mn 2p (b), Co 2p (c), and Ni 2p (d) XPS spectra of the CrMnCoNi aerogel.

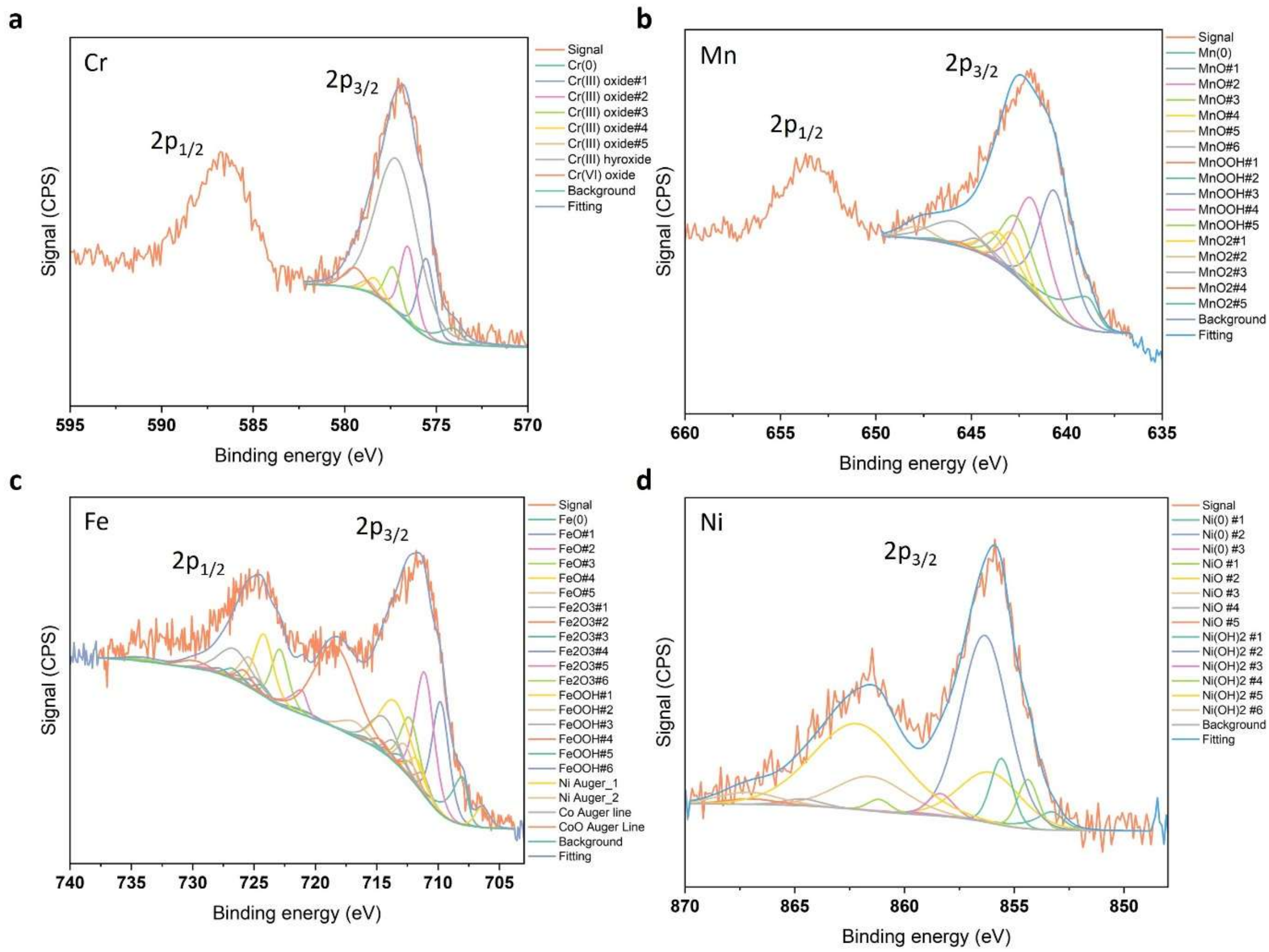

Figure S15 Deconvoluted Cr 2p (a), Mn 2p (b), Fe 2p (c), and Ni 2p (d) XPS spectra of the CrMnFeNi aerogel.

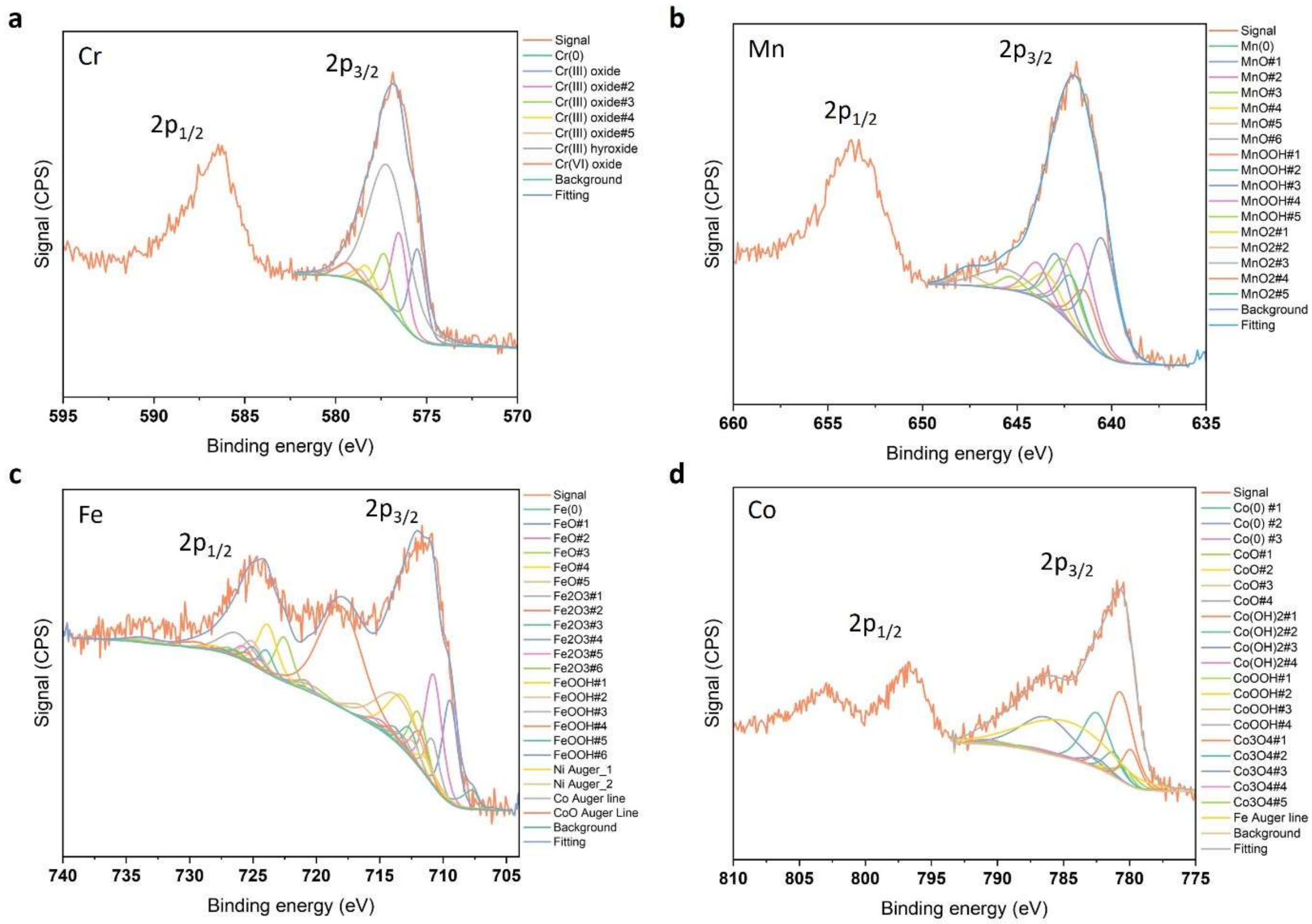

Figure S16 Deconvoluted Cr 2p (a), Mn 2p (b), Fe 2p (c), and Co 2p (d) XPS spectra of the CrMnFeCo aerogel.

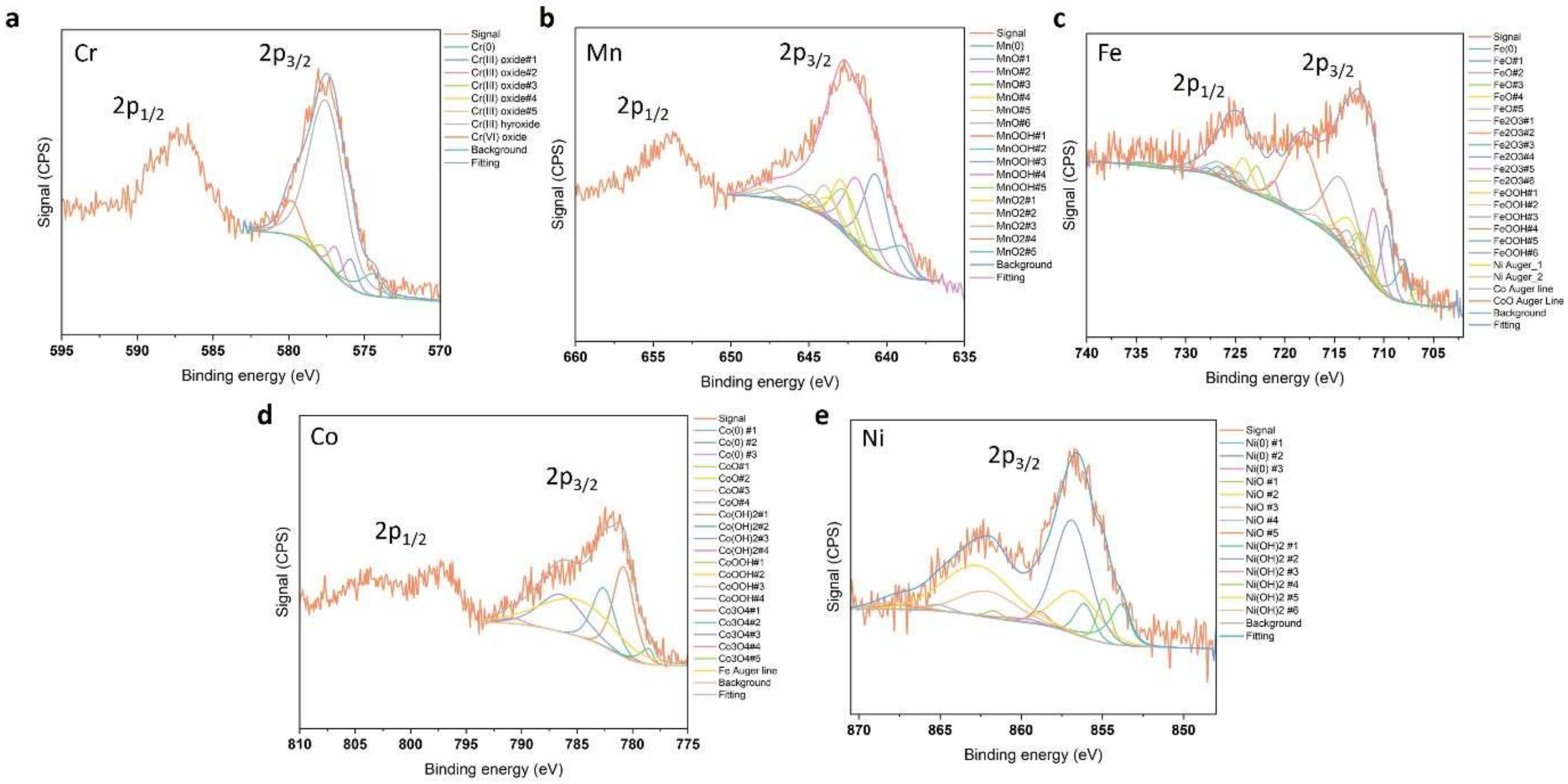

Figure S17 Deconvoluted Cr 2p (a), Mn 2p (b), Fe 2p (c), Co 2p (d), and Ni 2p (e) XPS spectra of the CrMnFeCoNi aerogel.

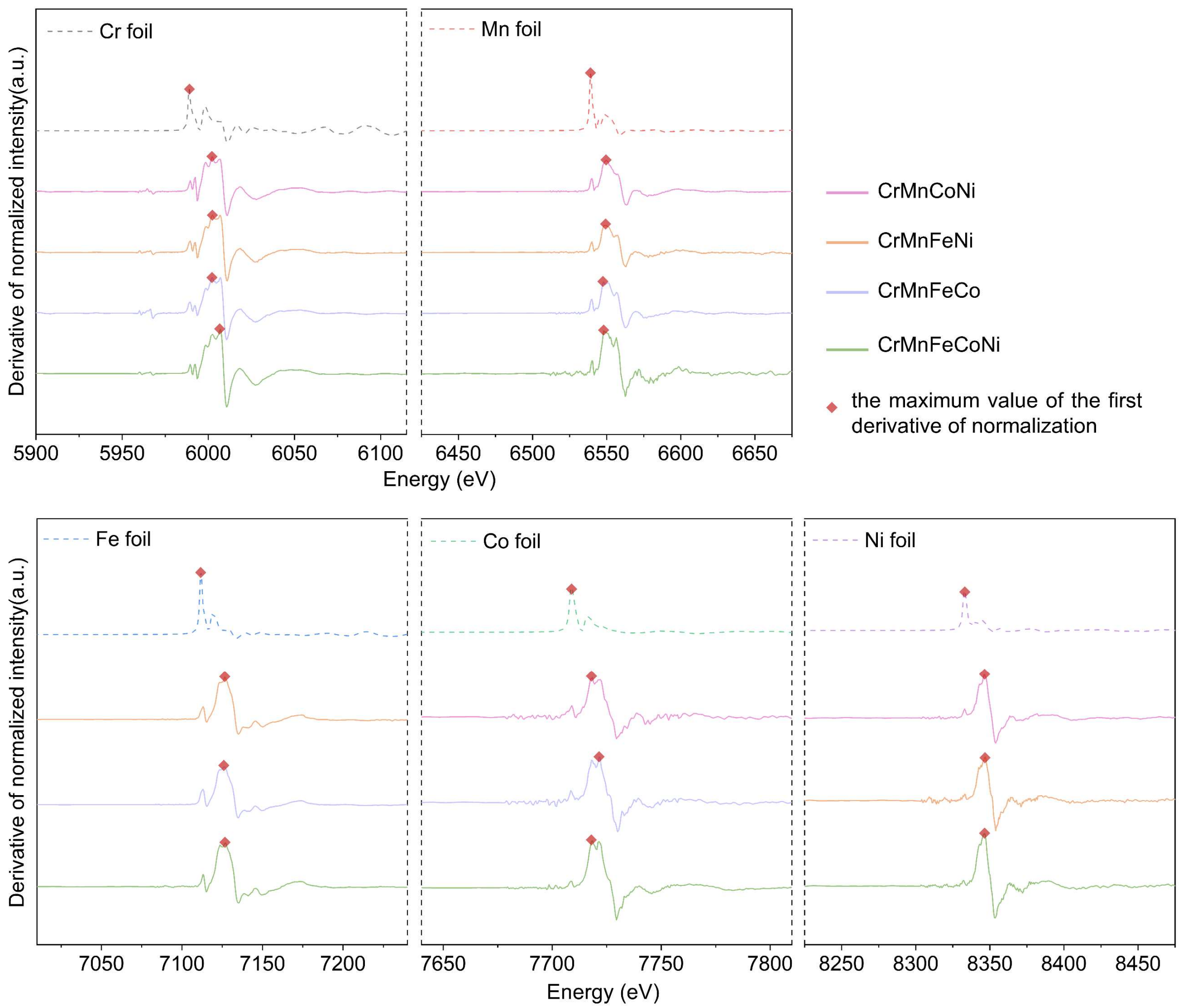

Figure S18 Maximum values of the first derivative of normalized XANES spectra for each element in metal foils, the as-prepared HEA and MEA aerogels.

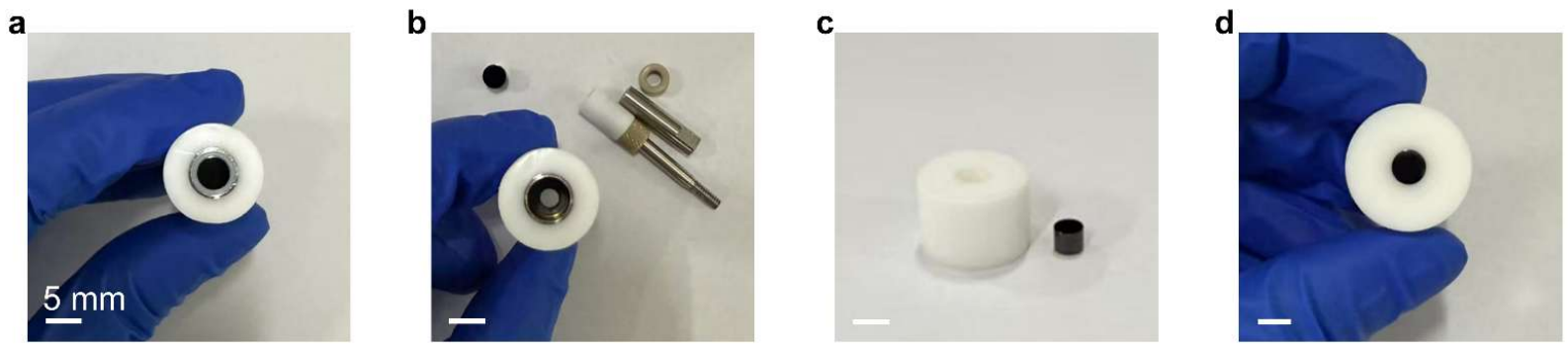

Figure S19 (a)The assembled and (b) disassembled RRDE, the polishing holder and a glassy carbon disk in the (c) disassembled and (d) assembled states. Scale bars represent 5 mm.

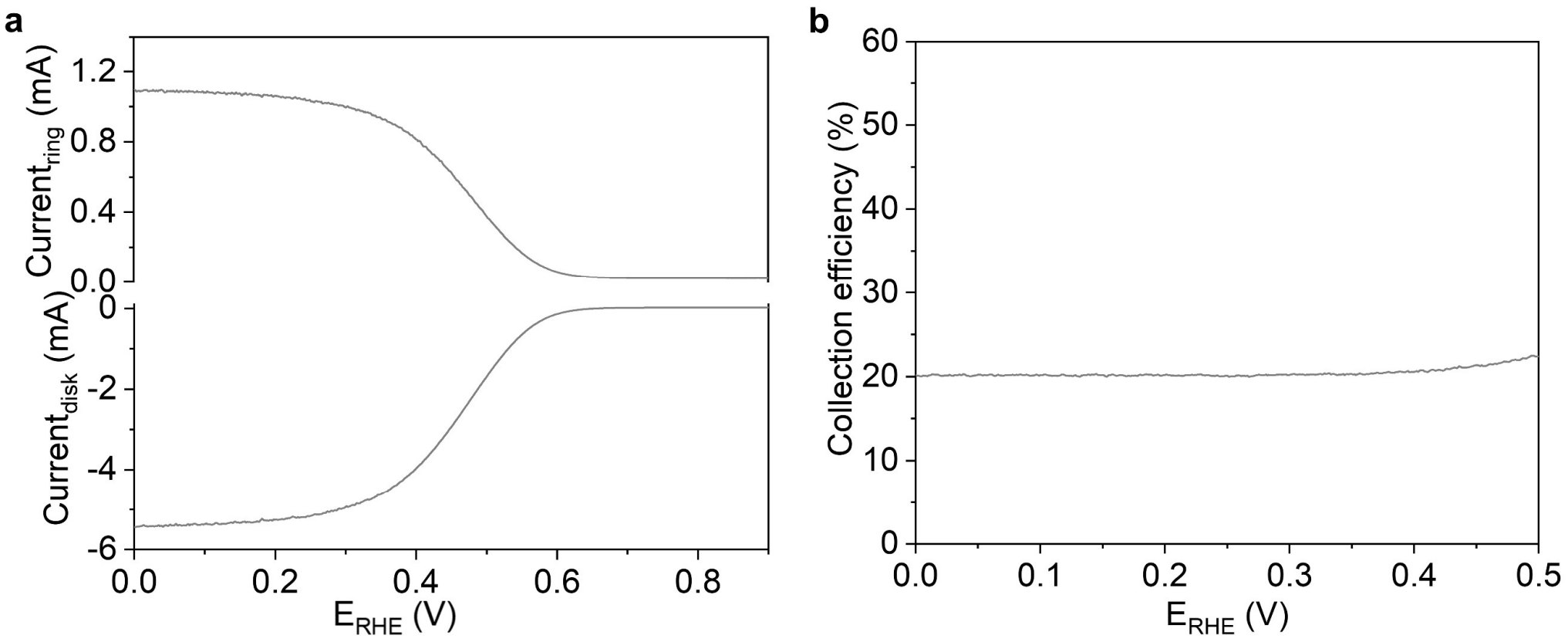

Figure S20 (a) Polarization curves of the blank RRDE electrode in an aqueous solution of 0.5 M Na_2SO_4 + 0.05 M $K_3Fe(CN)_6$ at 1600 rpm with a scan rate of 20 mV s^{-1}. (b) The calculated collection efficiency.

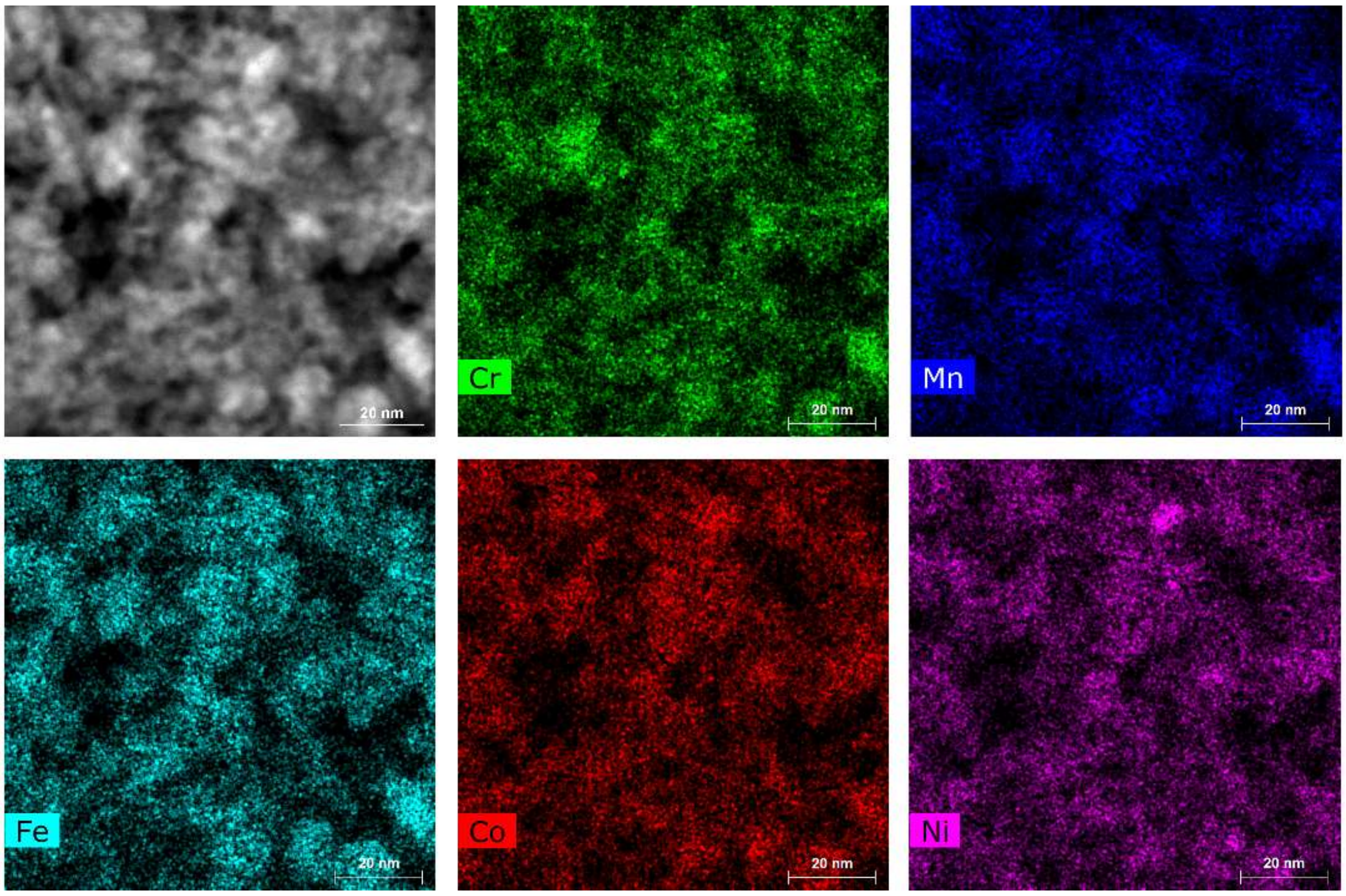

Figure S21 HAADF-STEM image and corresponding EDS-based element distribution maps of Cr, Mn, Fe, Co, and Ni in the CrMnFeCoNi HEA aerogel after 10k cycles accelerated durability test (ADT).

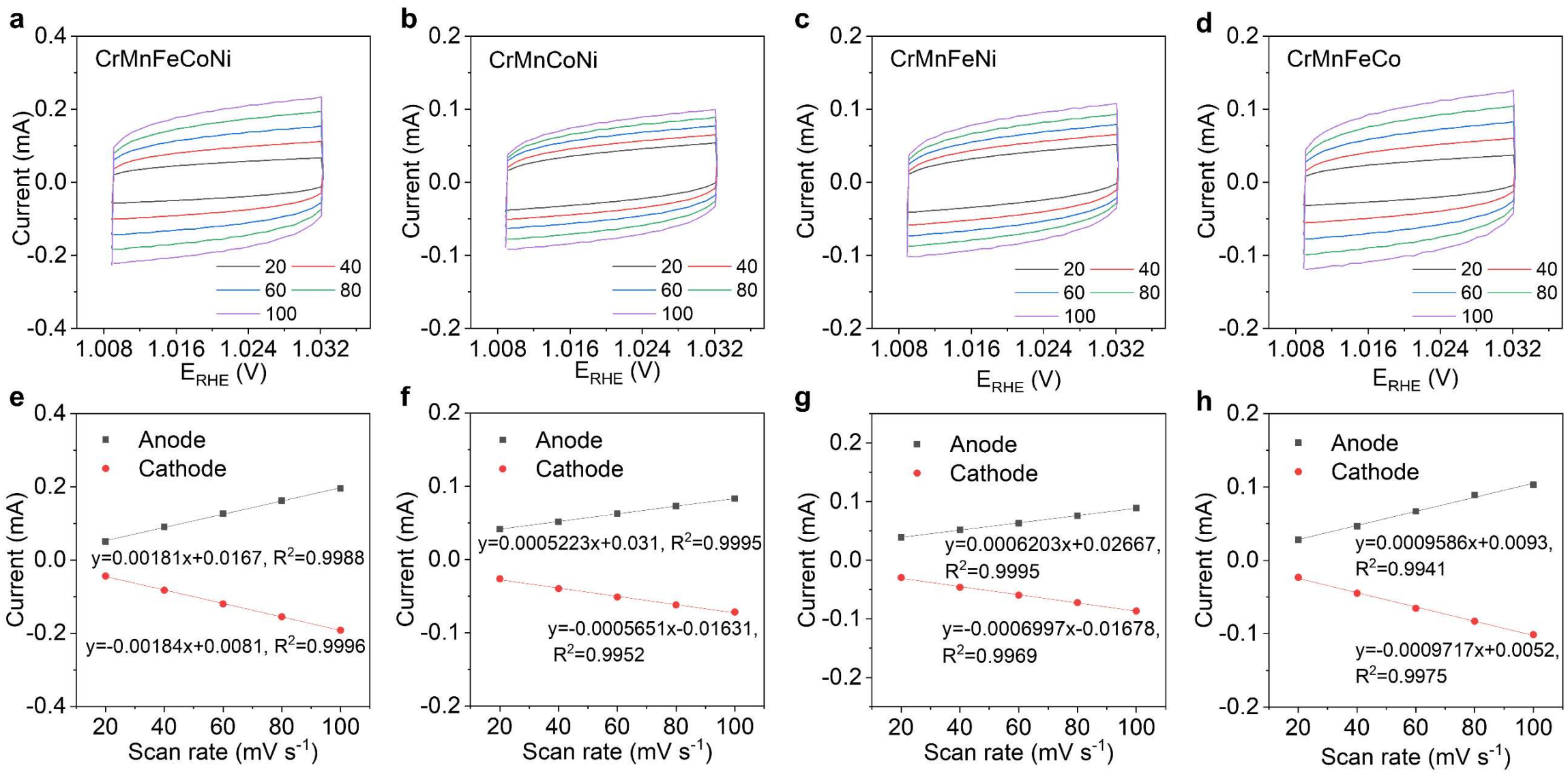

Figure S22 Double-layer capacitance measurements (C_{dl}) for determining the electrochemically active surface area (ECSA) of the resulting aerogels in 0.1 M KOH solution. (a-d) Cyclic voltammograms (CVs) obtained in the scan rate range from 20 to 100 mV s^{-1} in the non-Faradaic regions. (e-h) The corresponding plots of the anodic and cathodic current at 1.020 V_{RHE} against scan rate. (a and e), (b and f), (c and g) and (d and h) are CrMnFeCoNi, CrMnCoNi, CrMnFeNi, and CrMnFeCo aerogels, respectively.

Figure S22a-d shows the raw data for current versus scan rate plot, which were firstly obtained in N_2-saturated 0.1 M KOH solution at different scan rates (20, 40, 60, 80 and 100 mV s^{-1}). Subsequently, the anodic and cathodic currents at 1.020 V_{RHE} (a middle potential for the non-Faradaic region) were plotted versus scan rate, respectively (**Figure S22e-h**). After linear fitting, the double-layer capacitance (C_{dl}) was calculated by averaging the anodic and cathodic slopes (the unit is *"Farad"* (denoted as "F"), which are 1.83, 0.54, 0.67, and 0.97 mF for CrMnFeCoNi, CrMnCoNi, CrMnFeNi, and CrMnFeCo aerogels, respectively. Accordingly, the resulting ECSA was determined as follows[20-21]:

$$\frac{mA}{mV\ s^{-1}} = \frac{A \times s}{V} = \mathrm{F}$$

$$ECSA = \frac{C_{dl}}{C_s} = \frac{Slope_{(anode-cathode)}}{2C_s}$$

wherein the unit of the ECSA is *"cm²"*, and C_s represents the specific capacitance of the metal electrodes, which is 40 μF cm^{-2} for alkaline electrolytes. Taking CrMnFeCoNi aerogel as an example (**Figure S22**), $ECSA = \frac{Slope_{(anode-cathod\ )}}{2C_s} = \frac{0.00181+0.00184}{2\times 40} = 45.625\ cm^2$. Accordingly, ECSA values of CrMnCoNi, CrMnFeNi, and CrMnFeCo are calculated to be 13.59, 16.5, and 24.1 cm^2, respectively.

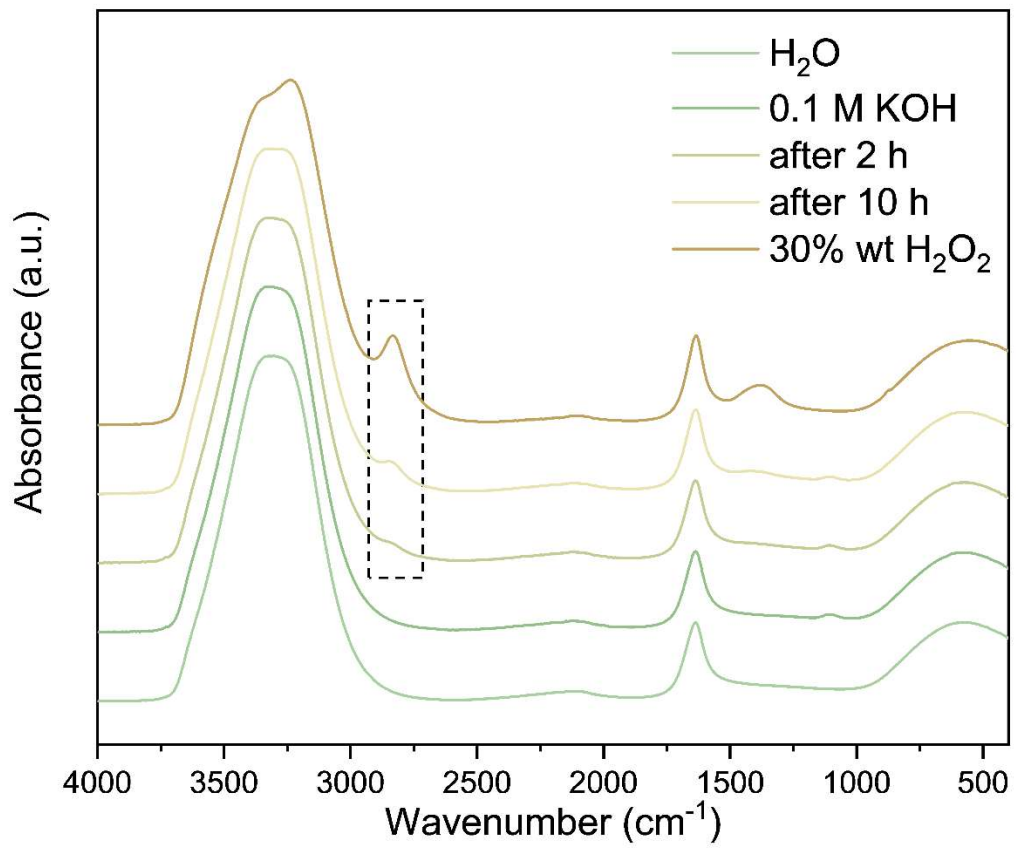

Figure S23 FTIR spectra of H_2O, 0.1 M KOH, the cathodic electrolyte after reaction at different reaction time, and 30% wt H_2O_2.

As shown in **Figure S23**, strong OH stretching vibration (at the range from 2900 to 3700 cm^{-1})[22] was observed in all analytes. After 2-hour electrolysis at an optimal working potential of 0.2 V_{RHE}, a small shoulder at 2760 cm^{-1} appeared and it became stronger after 10 hours, suggesting the H_2O_2 formation as verified by comparation with commercial 30% wt H_2O_2.

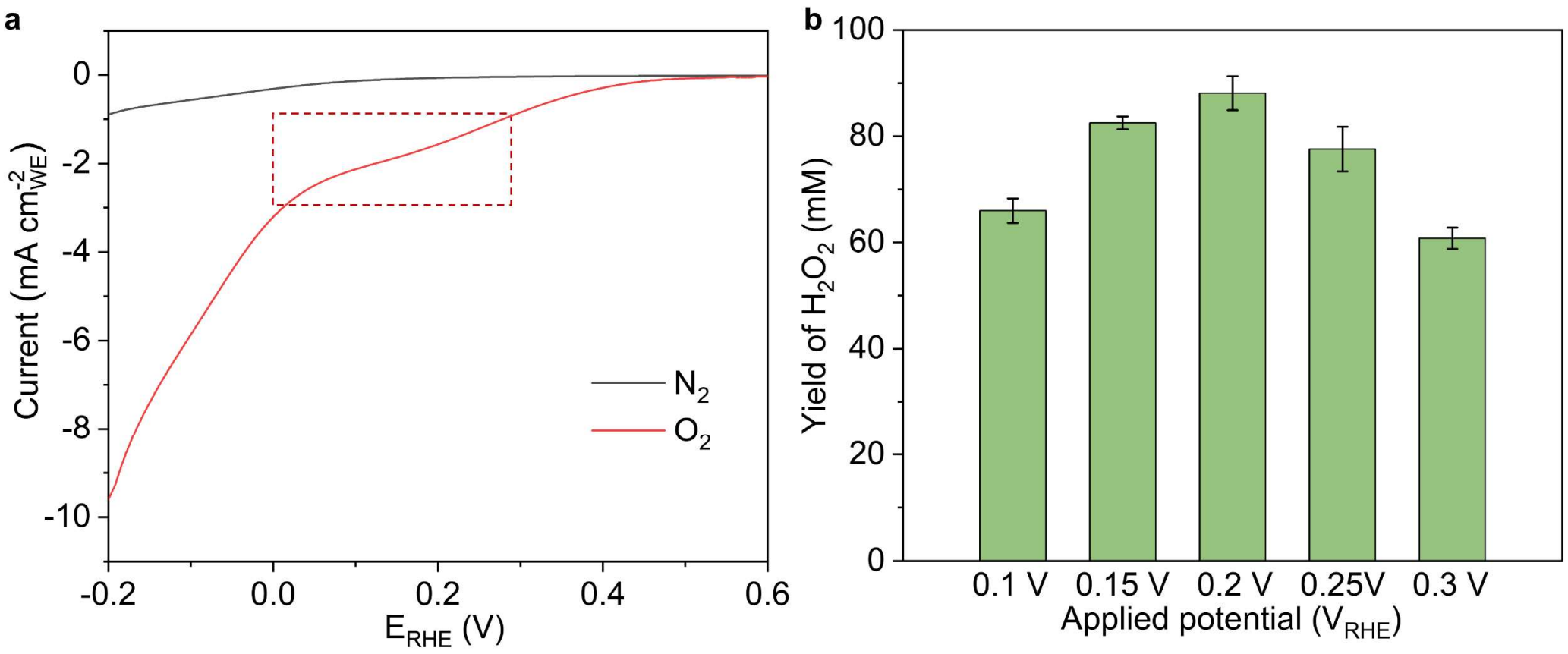

Figure S24 (a) LSV curves of CrMnFeCoNi HEA aerogels (HEAA) in the H-cell under N_2- and O_2-saturated 0.1 M KOH at a scan rate of 20 mV s^{-1}. "WE" in the y-axis label in (b) denotes the working electrode, which was a carbon cloth (1.0 × 1.5 cm). (b) The yield of H_2O_2 of the as-prepared CrMnFeCoNi HEA aerogel (HEAA) at different applied potentials (V_{RHE}), which corresponds to **Figure 4c**. Error bars in (b) represent standard deviation from three-time measurements.

The O_2-saturated LSV was recorded from -0.2 to 0.6 V_{RHE} to cover the full cathodic regime, including the ORR onset, the diffusion-limited region, and the potential range where HER may start to contribute. The chronoamperometry potentials were chosen within the ORR-dominated window (typically ≥ 0 V_{RHE}) to maximize H_2O_2 selectivity (**Figure S24a**). In our case, the electrolyte volume in cathode compartment was 40 mL, and the carbon cloth surface area was 1.5 cm^2. Therefore, the final concentration of generated H_2O_2 were calculated to be 66.0 mM at 0.1 V_{RHE}, 82.5 mM at 0.15 V_{RHE}, 88.1 mM at 0.2 V_{RHE}, 77.6 mM at 0.25 V_{RHE}, and 60.75 mM at 0.3 V_{RHE}, respectively (**Figure S24b**).

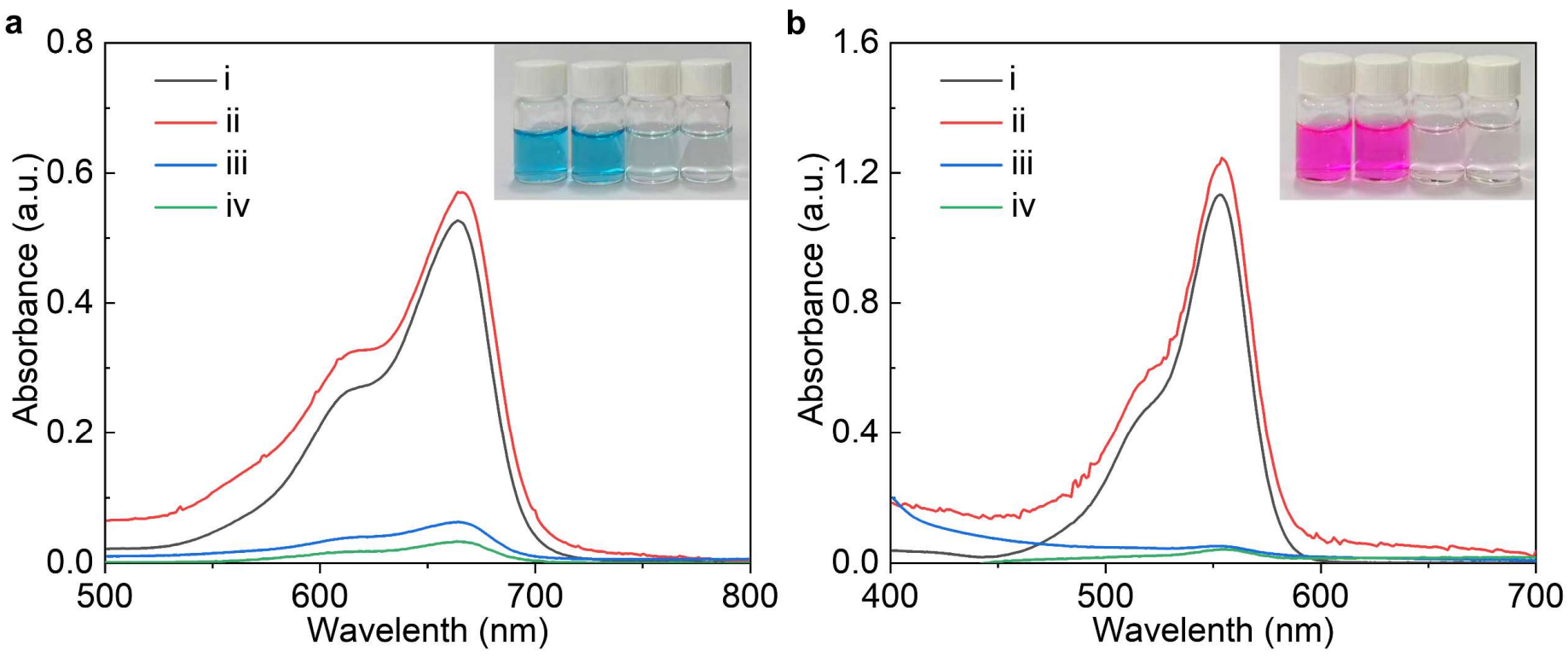

Figure S25 The UV-spectra and the corresponding photograph (inset) of (e) MB and (f) RhB decolorization by varying the presence of substrates, (i) MB or RhB, (ii) MB or RhB + H_2O_2, and MB or RhB + H_2O_2 + HEAA catalysts in (iii) dark and (iv) natural light condition.

The final absorbance of MB and RhB in the dark is slightly higher than that under ambient light, indicating no apparent light dependent behavior (i.e. no measurable photocatalytic contribution) of the CrMnFeCoNi aerogel.

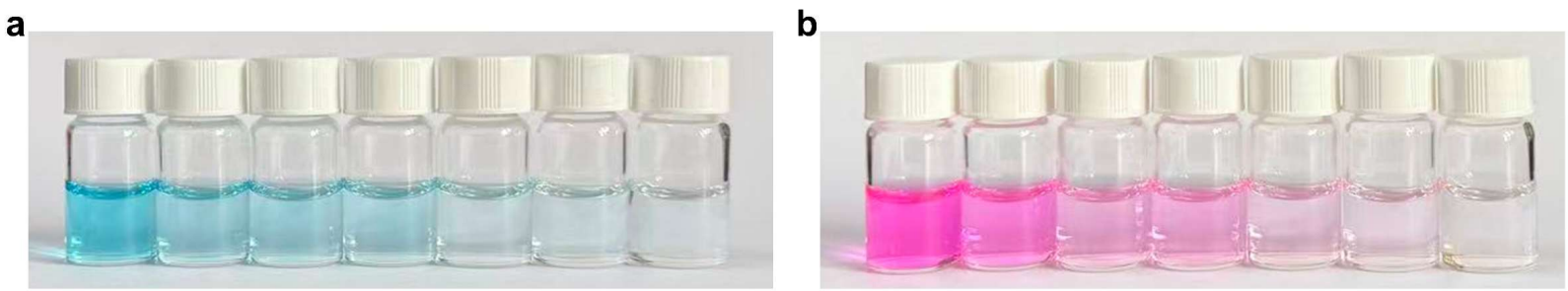

Figure S26 Photograph of MB and RhB decolorization at different times (corresponding to **Figure 4e-f**).

Tables

Table S1 Comparison of pore size analysis and BET surface area in the resulting aerogels.

	CrMnCoNi	CrMnFeNi	CrMnFeCo	CrMnFeCoNi		
	Water	Water	Water	Water	EtOH	Acetone
Pore Volume (cm^3 g^{-1})	0.189	0.177	0.342	1.087	0.112	0.454
Pore Diameter (nm)	3.6	3.6	3.6	4.1	4.1	22.6
BET surface area (m^2 g^{-1})	136.3	145.1	209.7	417.8	24.4	62.4

As shown in **Table S1**, the resulting aerogels are mesoporous materialswith a pore diameter in the range of 2-50 nm.

Table S2 Relative bulk element composition of the as-prepared aerogels obtained from SEM-EDS analysis.

Sample	Cr (at.%)	Mn (at.%)	Fe (at.%)	Co (at.%)	Ni (at.%)
CrMnCoNi	24.9	24.7	--	25.5	24.9
CrMnFeNi	23.3	25.1	29.6	--	22.0
CrMnFeCo	24.7	23.0	30.2	22.1	--
CrMnFeCoNi	20.1	19.6	22.7	18.9	18.7

Table S3 SEM-EDS area composition quantification of ball-milled micropowders.

Sample	Cr (at.%)	Mn (at.%)	Fe (at.%)	Co (at.%)	Ni (at.%)
CrMnCoNi	26.9	25.1	--	23.7	24.3
CrMnFeNi	22.4	24.9	30	--	22.7
CrMnFeCo	24.5	23.1	29.9	22.5	--
CrMnFeCoNi	19.9	19.5	23.3	18.7	18.6

Table S4 Relative element composition of the as-prepared aerogels obtained from ICP analysis.

Sample	Cr (at.%)	Mn (at.%)	Fe (at.%)	Co (at.%)	Ni (at.%)
CrMnCoNi	24.0	24.3	--	26.3	25.4
CrMnFeNi	23.0	24.1	29.6	--	23.3
CrMnFeCo	23.7	22.6	30.2	23.5	--
CrMnFeCoNi	19.8	19.9	22.0	19.2	19.1

Note that these values are the averages of three independent measurements.

Table S5 Constraints used during the peak fitting of all XPS spectra listed for each element.

Cr 2p	Cr^0		Cr(III) oxide (5 multiplets)		Cr(III) hydroxide		Cr(VI) oxide	
	min	max	min	max	min	max	min	max
Pos. Constr. / eV	574	574.4	575.5	579.1	577.1	577.5	479.4	479.8
FWHM Constr. / eV	0.7	1.1	0.8	1	2.5	2.7	1.28	1.48

Mn 2p	Mn^0		MnO (6 multiplets)		MnOOH (5 multiplets)		MnO_2 (5 multiplets)	
	min	max	min	max	min	max	min	max
Pos. Constr. / eV	638.5	639	640.2	645.9	641.1	645.5	642.4	646.8
FWHM Constr. / eV	1	1.3	1.5	1.95	1.05	1.45	1.05	1.45

Fe 2p	Fe^0		FeO (5 multiplets)		Fe_2O_3 (6 multiplets)		FeOOH (6 multiplets)	
	min	max	min	max	min	max	min	max
Pos. Constr. / eV	706.4	708	708.2	716.8	709.6	720.8	710.1	721.1
FWHM Constr. / eV	0.6	1	1.1	2.7	0.9	2.8	1.2	2.7

Co 2p	Co^0 (3 multiplets)		CoO (4 multiplets)		$Co(OH)_2$ (4 multiplets)		CoOOH (4 multiplets)	
	min	max	min	max	min	max	min	max
Pos. Constr. / eV	777.7	783.5	779.6	786.9	780	790.8	779.7	790.6
FWHM Constr. / eV	0.5; 2.8	0.9; 3.2	2.4	2.8	2.2	2.8	1.28	1.68

Co 2p							Co_3O_4 (5 multiplets)	
							min	max
Pos. Constr. / eV							779.2	789.9
FWHM Constr. / eV							1.1	1.7

Ni 2p	Ni^0 (3 multiplets)		NiO (5 multiplets)		$Ni(OH)_2$ (6 multiplets)			
	min	max	min	max	min	max		
Pos. Constr. / eV	851.6	859.8	852.7	867.5	853.9	867.6		

FWHM Constr. / eV	0.9; 2.28	1.1; 2.68	0.9; 3.1	1.1; 4.2	1.4; 2.85	1.7; 3.25

Table S6 Relative parameters derived from XAS data of each element in metal foils, the as-prepared HEA and MEA aerogels.

Sample	Element	E_{WL}	E_0	ΔE	ΔE_{WL}	WL intensity (a.u.)
metal foil	Cr	6009.06	5989.0	-	-	1.0333
	Mn	6557.0	6539.0	-	-	1.0501
	Fe	7131.55	7111.99	-	-	0.9979
	Co	7729.96	7709.0	-	-	0.9806
	Ni	8350.08	8333.0	-	-	0.9819
CrMnCoNi aerogel	Cr	6009.05	6002.5	13.5	-0.01	1.2806
	Mn	6560.0	6549.51	10.51	3.0	1.2342
	Co	7727.50	7718.28	9.28	-2.46	1.2939
	Ni	8351.43	8345.97	12.97	1.35	1.4007
CrMnFeNi aerogel	Cr	6009.02	6002.49	13.49	-0.04	1.3733
	Mn	6559.99	6549.52	10.52	2.99	1.0820
	Fe	7132.98	7126.49	14.5	1.43	1.2878
	Ni	8351.95	8346.95	13.95	1.87	1.3768
CrMnFeCo aerogel	Cr	6009.21	6007.01	18.01	0.15	1.3127
	Mn	6559.76	6547.98	8.98	2.76	1.2630
	Fe	7133.2	7126.46	14.47	1.65	1.2543
	Co	7727.48	7721.55	12.55	-2.48	1.3830
CrMnFeCoNi aerogel	Cr	6009.0	6007.02	18.02	-0.06	1.4523
	Mn	6559.51	6548.04	9.04	2.51	1.5813
	Fe	7132.99	7126.99	15	1.44	1.3475
	Co	7727.09	7718.59	9.59	-2.87	1.3967
	Ni	8350.50	8346.50	13.5	0.42	1.5704

Note that “WL” represents an abbreviation for “white line”.

Determination of E_0, white-line position E_{WL}, and white-line intensity. E_0 was defined as the energy at the maximum of the first derivative of the normalized XANES spectrum[7] (**Figure S18**). The white-line position E_{WL} was taken as the energy of the maximum in the white-line region after edge-step normalization. All spectra were energy-referenced to the corresponding metal foils. **Table S6** summarizes E_0, E_{WL}, the relative shifts (ΔE and ΔE_{WL}, see behow), and the normalized white-line intensity.

$$\Delta E = E_{sample} - E_{foil}$$

$$\Delta E_{WL} = E_{WL,sample} - E_{WL,foil}$$

Notably, Mn exhibits a consistent positive white-line shift for all aerogels ($\Delta E_{WL} = +2.5\ to + 3.0\ eV$), whereas Co shows a negative shift ($\Delta E_{WL} = -2.46\ to - 2.87\ eV$). The large ΔE values primarily reflect the operational definition of and are therefore discussed as relative descriptors rather than direct measures of oxidation-state changes.

Table S7 A summary of repeatability in RRDE tests at the range of 0-0.5 V_{RHE}.

Sample	H_2O_2 selectivity (%)	n
CrMnCoNi	53.9±0.3	3.0±0.03
CrMnFeNi	38.0±0.4	3.2±0.04
CrMnFeCo	53.9±0.8	3.0±0.05
CrMnFeCoNi	95.6±0.6	2.1±0.03

Note that the value is an average from three measurements.

To keep the result concise while ensuring transparency, a summary of repeatability was provided (**Table S7**), reporting the mean ± standard deviation from three independent measurements for the key metrics (e.g., H_2O_2 selectivity, and electron-transfer number (n)). The RRDE used in this work (series No. AFE6R1PT, Pine Research Instrument) consists of a detachable glassy carbon disk and a fixed Pt ring (**Figure S19**). Therefore, repeatability was evaluated by independently preparing catalyst layers on the same glassy carbon disk (**Figure S19a-b**). After each test, the glassy carbon disk was removed from the RRDE assembly using the specific change disk toolkit (AFE6K050), transferred to a particular polishing holder (see **Figure S19c-d**), and re-polished following the same protocol (see the *Electrochemical test* section in the SI). The disk was then reinstalled into the RRDE, a freshly prepared catalyst ink was drop-cast, and the RRDE measurement was repeated under identical conditions. The variations are relatively small, supporting good reproducibility of the reported performance."

References

[1] N.F. Shkodich, T. Smoliarova, H. Ali, B. Eggert, Z. Rao, M. Spasova, I. Tarasov, H. Wende, K. Ollefs, B. Gault, M. Farle, Acta Mater. **2025**, *284*, 120569.

[2] C. Wang, J. Herranz, R. Hübner, T. J. Schmidt, A. Eychmüller, *Acc. Chem. Res.* **2023**, *56*, 3, 237–247.

[3] D. A. Shirley, *Phys. Rev. B* **1972**, *5*, 4709.

[4] M. C. Biesinger, *Appl. Surf. Sci.* **2022**, *597*, 153681.

[5] C. D. Wagner, W. M. Riggs, L. E. Davis, J. F. Moulder, G. E. Muilenberg, Handbook of X-ray photoelectron spectroscopy, Perkin-Elmer Corporation, Eden Prairie, Minnesota, USA, **1979**.

[6] S.-M. Yang, L.-L. Guo, B. Nan, Y. Zhao, Y.-Q. Wu, Z. Guo, C. Tian, B. Zhao, C.-F. Xue, J. Zhao, S. Song, Z.-Y. Liang, L.-N. Li, Y. Wang, R.-Z. Tai, *Nucl. Sci. Tech.* **2026**, *37*, 11.

[7] B. Ravel, M. Newville, *J. Synchrotron Rad.* **2005**, *12*, 537–541.

[8] J. P. Perdew, K. Burke, M. Ernzerhof, *Phys. Rev. Lett.* **1996**, *77*, 3865.

[9] S. Goedecker, M. Teter, J. Hutter, *Phys. Rev. B* **1996**, *54*, 1703.

[10] S. Grimme, J. Antony, S. Ehrlich, H. Krieg, *J. Chem. Phys.* **2010**, *132*, 154104.

[11] X. Zhang, C. Wang, K. Chen, A. H. Clark, R. Hübner, J. Zhan, L. Zhang, A. Eychmüller, B. Cai, *Adv.Mater.* **2023**, *35*, 2211512.

[12] J. Chen, Q. Ma, X. Zheng, Y. Fang, J. Wang, S. Dong, *Nat. Commun.* **2023**, *13*, 2808.

[13] C. Zhang, W. Shen, K. Guo, M. Xiong, J. Zhang, X. Lu, *J. Am. Chem. Soc.* **2023**, *145*, 11589–11598.

[14] H. R. Byon, J. Suntivich, Y. Shao-Horn, *Chem. Mater.* **2011**, *23*, 3421–3428.

[15] Y. Zhang, Z. Lyu, Z. Chen, S. Zhu, Y. Shi, R. Chen, M. Xie, Y. Yao, M. Chi, M. Shao, Y. Xia, *Angew. Chem., Int. Ed.* **2021**, *133*, 1–6.

[16] X. Zhang, T. Wang, C. Wang, R. Hübner, A. Eychmüller, J. Zhan, B. Cai. *Small* **2023**, *19*, 2207557.

[17] D. Zhang, C. Zhang, J. Liu, Q. Chen, X. Zhu, X.C. Liang, *ACS Appl. Nano Mater.* **2019**, *2*, 28–39. Fromme, T.; Reichenberger, S.; Tibbetts, K. M.; Barcikowski, S. *Beilstein J. Nanotechnol.* **2024**, *15*, 638–663.

[18] R. Du, J. Wang, Y. Wang, R. Hübner, X. Fan, I. Senkovska, Y. Hu, S. Kaskel, A. Eychmüller *Nat. Commun.* **2020**, *11*, 1590.

[19] J. Johny, Y. Li, M. Kamp, O. Prymak, S.-X. Liang, T. Krekeler, M. Ritter, L. Kienle, C. Rehbock, S. Barcikowski, S. Reichenberger, *Nano Res.* **2022**, *15*, 4807–4819.

[20] S. S. Jeon, P. W. Kang, M. Klingenhof, H. Lee, F. Dionigi, P. Strasser, *ACS Catal.* **2023**, *13*, 1186–1196.

[21] M. A. Ehsan, A. Khan, A. S. Hakeem, *ACS Appl. Energy Mater.* **2023**, 6, 9556–9567.

[22] A. D. Ninno, A. C. Castellano, E. D. Giudice, *J. Phys.: Conf. Ser.* **2013**, *442*, 012031.
